# Establishing Peer Recovery Support Services to Address the Central Appalachian Opioid Epidemic: The West Virginia Peers Enhancing Education, Recovery, and Survival (WV PEERS) Pilot Program

**DOI:** 10.13023/jah.0303.04

**Published:** 2021-07-25

**Authors:** Stephen M. Davis, Amanda N. Stover, Herb Linn, Jon Dower, Daniel McCawley, Erin L. Winstanley, Judith Feinberg

**Affiliations:** School of Public Health, West Virginia University; School of Pharmacy, West Virginia University; School of Public Health, West Virginia University; West Virginia Sober Living; School of Medicine, West Virginia University

**Keywords:** Appalachia, peer recovery support services, opioid use disorder, addiction, opioids, overdose, rural, central Appalachia

## Abstract

**Introduction:**

Central Appalachia has been disproportionately affected by the opioid epidemic and overdose fatalities. We developed West Virginia Peers Enhancing Education, Recovery, and Survival (WV PEERS), a program based on peer recovery support, to engage individuals using opioids and link them with a range of services.

**Methods:**

Community partners providing services to individuals with opioid use disorder (OUD) were identified and collaborations were formalized using a standardized memorandum of understanding. The program was structured to offer ongoing peer recovery support specialist (PRSS) services, not just a one-time referral. A website and cards describing the WV PEERS program were developed and disseminated via community partners and community education sessions.

**Results:**

Overall, 1456 encounters with individuals with OUD (mean= 2 encounters per individual) occurred in a variety of community settings over 8 months. The majority of referrals were from harm reduction programs. Overall, 63.9% (n=931) of individuals served by WV PEERS accessed services for substance use disorders and/or mental health problems. Over half (52.3%; n = 487) of individuals entered substance use and/or mental health treatment, and nearly a third (30.4%; n = 283) remained in treatment over six months.

**Implications:**

Using the WV PEERS model, PRSSs effectively engaged and linked individuals with OUD to mental health and substance use treatment in rural central Appalachia. Future research is needed to determine whether these services reduce the risk of overdose mortality.

## INTRODUCTION

Since the 1980s, people with a history of mental illness, addiction, and/or trauma have become increasingly involved in systems of care as peer supporters for people with behavioral health conditions.^1^ The Substance Abuse and Mental Health Services Administration (SAMHSA), the U.S. federal agency responsible for behavioral health services, defines a peer worker as “a person who uses his or her lived experience of recovery from mental illness and/or substance use disorder, plus skills learned in formal training, to deliver services in behavioral health settings to promote mind–body recovery and resiliency”.^2^ Numerous studies of peer support for individuals with substance use disorders (SUD) demonstrate improved relationships with providers and social supports, increased referrals to treatment, increased satisfaction with the treatment experience overall, reduced rates of relapse, and increased retention in treatment.^3^–^15^ Peer support services are critical for people not formally engaged in behavioral health services. Peer supporters serve as models for a life in recovery, which may motivate clients to achieve and sustain their own recovery.^5^

Despite these positive findings, the majority of these peer support studies^3^–^13^ were conducted in urban areas that have lower overdose fatality rates^16^ and greater access to behavioral health services compared to rural Appalachia.^17^ According to the Centers for Disease Control and Prevention (CDC), for each year from 2010 through 2018, rural West Virginia (WV), which is the only state located entirely within Appalachia, experienced the highest rate of fatal drug overdoses (OD) in the U.S.^18^ The age-adjusted fatality rate for WV peaked at nearly 58 deaths per 100,000 people in 2017 and remained the highest in the country at 52.8 per 100,000 in 2019.^18^

According to the WV Office of Drug Control Policy, WV recorded 871 OD deaths from all drugs in 2019 with 76.8% involving at least one opioid.^19^ However, despite this health disparity, availability of harm reduction and other health services in rural areas is limited due to differing social norms that impacts laws and policies governing these services.^20^ Therefore, a pilot study was conducted—the West Virginia Peers Enhancing Education, Recovery, and Survival (WV PEERS), to determine if support from people state-certified as peer recovery support specialists—referred to as Peer Recovery Coaches (PRCs)—results in improved rates of subsequent contact with and utilization of a range of substance use and mental health services. The target population included (1) opioid OD survivors or individuals with opioid use disorder (OUD) who were treated in the West Virginia University (WVU) J.W. Ruby Hospital Emergency Department (ED); (2) people with SUD referred by social services and other agencies in Monongalia County WV; and (3) individuals who self-referred for assistance and support.

## MATERIALS AND METHODS

### STUDY POPULATION

Monongalia County, the location of the City of Morgantown and the main campus of WVU, experienced an unprecedented 82% increase in OD deaths from all drugs from 2015 to 2016, from 28 to 51 deaths, and a 78% increase in opioid-related overdose deaths, from 23 to 41 deaths.^19^ Among all 55 counties, Monongalia County experienced the fourth highest number of opioid-related overdose deaths in 2016. The dramatic increase in OD deaths in Monongalia County in 2016 caught the attention of local officials and spurred them to seek evidence-based solutions to the drug problem.

### STRUCTURE OF THE INTERVENTION

**Figure 1:**
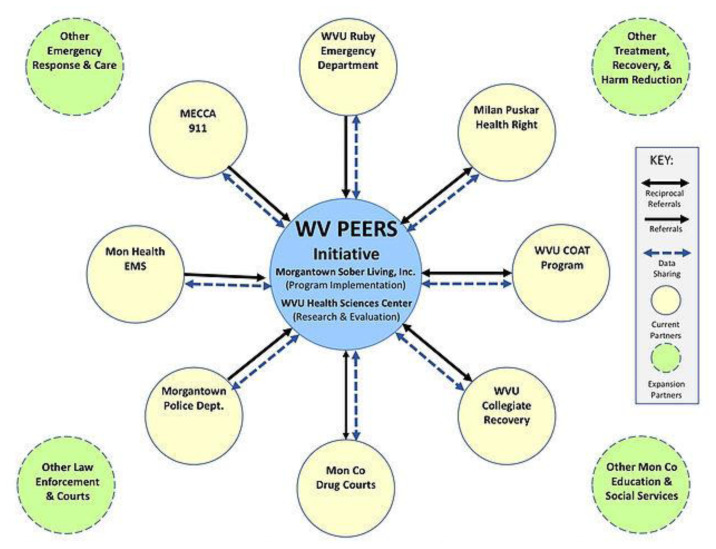
West Virginia Peers Enhancing Education, Recovery, and Survival Community Partners

In 2017–2018, the WV PEERS program established standardized memoranda of understanding with a variety of community partners including the Ruby Hospital ED, the Morgantown Police Department, county drug courts, a local syringe services program, educational institutions and social services in Monongalia County, West Virginia (Figure 1). The study began enrolling participants in April 2018.

#### Peer Recovery Coaches Certification and Training Requirements

West Virginia PEERS PRCs were certified by the West Virginia Certification Board for Addiction & Prevention Professionals (https://www.wvcbapp.org/). Certification requires a defined course of study, 500 hours of experience including 25 hours of direct supervision, and at least 2 years of continuous sobriety. PRCs were hired and trained by Morgantown Sober Living, Inc. A policies and procedures manual was developed to standardize methods and materials. Training, coordination, and oversight were provided by an experienced PRC supervisor who also served as the WV PEERS Community Outreach Coordinator, and counselling arrangements ensured that the study did not impair the PRCs’ own recovery.

#### Study Procedures

**Figure 2:**
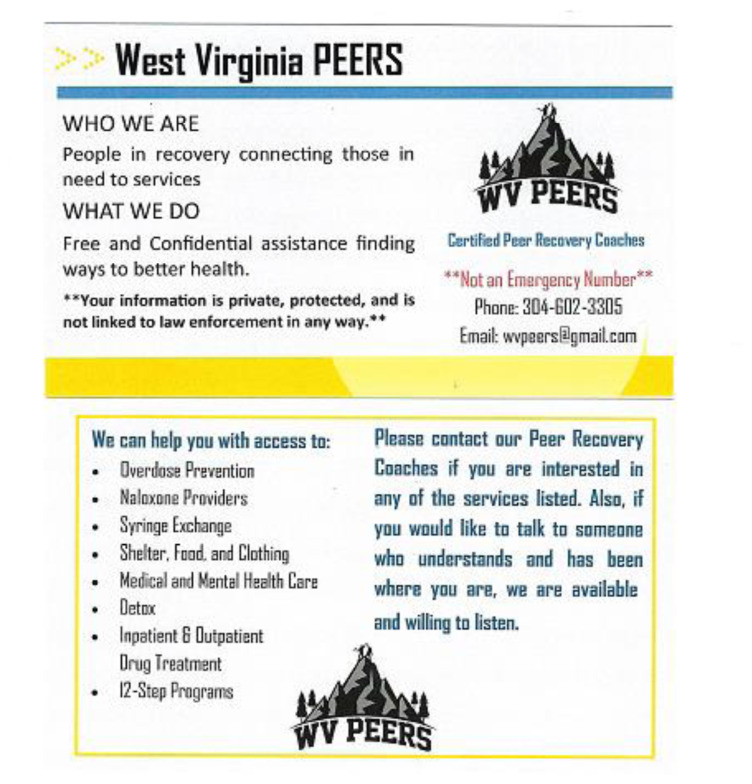
WV PEERS Leave-Behind Cards

Upon engagement, PRCs (1) provided OD prevention education and access to naloxone; (2) assessed interest in accessing an addiction treatment program, medical and/or mental health care; (3) assessed interest in accessing the local syringe services program; and (4) provided follow-up contact to determine possible recurrent overdose, naloxone use, and to continue to offer linkage to prevention and treatment services. Individualized coaching was guided by a motivational interviewing framework. Individuals who did not provide consent for the study were given an IRB-approved card describing WV PEERS services and contact information in case they changed their mind (Figure 2). While the program emphasized preventing future ODs and decreasing OD deaths, it also included a range of medical and social services, such as SUD treatment, mental and physical health care, as well as access to shelter, food, or childcare, and was structured to respond to the participant’s expressed hierarchy of needs rather than a narrow emphasis on OD prevention. This study was approved by the West Virginia University Institutional Review Board.

West Virginia PEERS services were divided into six categories: substance use, harm reduction, infectious diseases, mental health, primary medical care, and social services. The PRC discussed options for which they could provide linkage within each category that were tailored to address the client’s hierarchy of needs and were based on community availability. Clients’ hierarchy of needs was based on Abraham Maslow’s five-tiered approach to needs of humans. These tiers include physiological, safety, love, esteem, and self-actualization, requiring each tier’s needs to be satisfied prior to ascending to the next tier.^21^ PRCs integrated Maslow’s concept that an individual with a substance use disorder cannot adequately address a mental health condition without physiological needs (food, shelter) being fulfilled to ensure that participants were matched to appropriate services.

Utilizing encrypted tablets connected to REDCap as a secure data collection tool, PRCs were able to enter data remotely when engaging and tracking vulnerable populations, using an IRB approved informed consent. The specific survey questions are available as an online supplement (see Additional File).

Peer Recovery Coaches recontacted clients at 1 and 3 months after their initial visit to assess whether the client had accessed the services to which they had been linked. At each follow-up contact, the PRC re-assessed the client’s interest in service referral(s) and all referrals were documented in the study database.

Linkages were defined as communication between a third-party provider and the client; linkages may have included services provided by a community stakeholder. These linkages were reported by clients at follow-up or between data collection intervals if the client reached out to a peer specialist for support services.

#### Referral Avenues

Participants were referred to WV PEERS by the treating physician in the ED or by established partners based on their clients’ reported needs. The inclusion criteria for the WV PEERS pilot program evaluation were: (1) having experienced a nonfatal opioid overdose in Monongalia County, (2) at least 18 years old, (3) English-speaking, and (4) cognitive ability to understand and willingness to provide written informed consent. In addition, any individual with a SUD could be referred by a local agency or could self-refer for support and potential linkage to a range of services. A PRC was located in the ED during shifts identified as having higher OD rates based on a review of ED admissions, and were on-call during other time periods to respond to a new ED admission for OD. A website was developed that provided detailed information on the WV PEERS program (https://www.wvpeers.com/), as well as direct contact information for PRC services.

The second mode of referral was initiated by a collaborating community agency. For example, following the Law Enforcement Assisted Diversion (LEAD) program developed in Seattle,^22^ the Monongalia County drug court adopted a pre-booking diversion program that allows officers to redirect low-level offenders engaged in drug use to community-based services instead of jail and prosecution. PRCs worked directly with and provided support for referred offenders to obtain and remain in SUD treatment, and a WV PEERS case manager was available for assistance with housing and other needs. Lastly, people with SUD could self-refer to WV PEERS.

Another program element focused on outreach efforts critical to combatting the opioid crisis at a community level. Led by the Community Outreach Coordinator, WV PEERS staff conducted a targeted, sophisticated effort to reach Monongalia County residents to deliver education about SUD as a chronic, relapsing brain disease, the local resources available for people with SUD, and information on WV PEERS services. Our outreach efforts utilized a combination of public information sessions and social media to alert healthcare providers, lawyers, police, educators other professionals and community members about WV PEERS services and how to access them. As part of this effort, PRCs were available to speak about SUD and the risk of OD in secondary and post-secondary educational settings, community meetings, church functions, and other venues on request.

#### Statistical Analysis

Descriptive statistics were calculated on the overall number of referrals, referral sources, proportions of individuals engaging in substance misuse and/or mental health treatment as well as treatment entry, retention, and completion.

## RESULTS

Between April and December 2018, WV PEERS PRCs received 1456 referrals for services with an average of 2 encounters per individual (Table 1). The majority of referrals (62.5%) were from local harm reduction services and other community stakeholders. An additional one-third came from social service agencies and self-referral by individuals using the contact information on distributed cards and the website. A small minority came from first responders, the judicial system, and schools. Among all 1456 encounters, 63.9% (n=931) were linked to treatment for substance use and/or mental health. Slightly over half (52.3%; n = 487) of all encounters completed treatment for substance use and/or mental health, and nearly a third (30.4%; n = 283) remained in treatment for at least six months (Table 1).

Although schools represented less than 1% of all referrals, 100% of participant encounters resulted in treatment for substance use and/or mental health, with 7 of 10 remaining in treatment for at least six months. Individuals referred by the drug courts were also highly likely to engage in treatment (8 of 10 encounters) with half remaining in treatment for at least six months (Table 1).

West Virginia PEERS delivered 38 public information sessions about SUD and services offered by the program in a wide range of settings including schools, community meetings, churches, and other community locations (Table 2).

## DISCUSSION

A PRC–led program to provide support and warm handoffs to treatment and other services for individuals with SUD was successfully implemented in a rural central Appalachian county significantly impacted by the opioid crisis. PRCs— people with lived experience of OUD now in long-term recovery—met individuals with SUD in a variety of community settings and successfully linked them to a range of resources to meet their expressed needs, from social services to treatment for SUD and mental health issues.

Peer Recovery Coaches have been shown to be successful in a variety of settings.^5^,^8^ Previous research has shown that when peer support is available, patients are less likely to need future hospitalization and/or crisis services.^23^,^24^ Peer-delivered services have also been shown to improve social functioning and quality of life and to reduce substance use.^24^ Similarly, studies have shown that having a peer on the treatment team increases positive outcomes, including fewer and shorter hospitalizations and the ability to function in the community without utilizing mental health services.^25^ However, pooled treatment effects are challenged by the wide variability in the definitions of a peer recovery support specialist with varying levels of required training and certification. In particular, there is a dearth of literature and research on the effectiveness of this approach in outpatient and residential settings.^8^ Therefore, it is notable that we report the success of this approach with linkage to treatment implemented in outpatient and residential settings in a nonurban setting. Our linkage rate was almost double the rate observed in a recent mobile outreach program for opioid overdose survivors in Houston, TX (63.9% versus 33%, respectively).^26^ Although the specific factors contributing to our higher linkage rate merit further investigation, most PRSS programs reported in the published literature are located in urban areas, and the higher rates of linkage to services observed in this study may be associated with it being located in a rural area, which may have smaller close-knit social relationships compared to urban settings.

Transportation arrangements made by our peers to treatment sites may also have impacted our linkage rates. A previous study in our rural Appalachian environment reported a much higher linkage to treatment for individuals screening positive for HIV and HCV in an academic emergency department than in other urban settings, in part due to the ability of peer navigators to facilitate transportation to follow-up appointments.^27^ However, one previous study in a rural location highlighted the difficulty of identifying PRCs available in the rural setting and the transportation difficulties that affected their ability to engage individuals with SUD.^28^ These transportation difficulties suggest the potential role of peer recovery support services delivered via telehealth in rural areas.^28^,^29^ Although many transportation difficulties were not identified in this pilot study, factors that uniquely influence the successful implementation of peer services in central Appalachia and other rural areas merit further investigation. Studies examining specific factors affecting different treatment retention rates by referral source are also needed. Such factors may include variability in provider levels of experience and specialization and variability in client stages of change.

Additional information is also needed on the overall quality and cost-effectiveness of this approach to promote sustainability and formal incorporation of these services into the West Virginia Medicaid program. More specifically, West Virginia Medicaid recently implemented a 1115 Substance Use Disorder (SUD) Waiver. This cost-neutral waiver provides reimbursement for methadone and peer recovery support services and expansion of residential treatment services.^30^ Reimbursement for peer recovery support services is limited to established patients treated at licensed behavioral health agencies and so would not have covered the services provided by WV PEERS.^30^ However, information from our pilot will be useful to the formal evaluation of the 1115 SUD Waiver, which the state is currently undertaking with academic partners. Information from this evaluation will be shared with state officials who will be tasked with making decisions concerning the formal adoption of 1115 SUD waiver elements, including peer recovery support services, into the WV Medicaid program. Importantly, although cost-effectiveness is one component of this evaluation, equally important to the state is the quality and acceptability of the peer services to both individuals with substance use disorder and treatment providers. SAMSHA also advocates for policy changes that include peer support services in the treatment process. These services are a key component of the Trauma-Informed Care Model, which emphasizes involving patients in the treatment process. Evidence suggests that patients are more likely to be involved in the treatment process when interacting with peers who have lived experience.^31^

### Limitations

Although we were able to effectively implement PRC support in a local ED and other outpatient settings directly affected by the opioid epidemic, these results may not be generalizable to other EDs and outpatient settings in West Virginia or other rural communities. Additional information is needed to assess the long-term impact on successful engagement and linkage to treatment, reducing the rate of opioid ODs, and the cost-effectiveness of peer-provided services.

## IMPLICATIONS

West Virginia Peers Enhancing Education, Recovery, and Survival engaged individuals with SUD in nonurban outpatient and community settings in Monongalia County, West Virginia, generally resulting in successful linkage to substance use treatment and mental health services. The model is likely amenable to replication in other rural areas of Central Appalachia. Communities interested in following the WV PEERS model should consider an approach to engaging and gaining the support of community stakeholders similar to that taken in Monongalia County. First, a small group of academic- and community-based collaborators convened a multisector, multidisciplined planning team comprising participants from public health (especially injury prevention), emergency medicine, SUD treatment and recovery, and community justice (particularly county drug courts). Then, the planning team conducted a community meeting to present and discuss peer recovery support strategies among a broad set of stakeholders (adding leaders from EMS, the faith-based community, harm reduction, law enforcement, local media, and state and local government, among others). Subsequently, planners facilitated discussions with leaders in key stakeholder organizations to garner their support and participation and build on or create relationships centered on collaborative care and support of patients, clients, and offenders with SUD. Perhaps the key facilitating factor was early identification of a strong, local recovery program committed to integrating WV PEERS into its mission. Future research should elucidate the specific factors associated with successful retention in substance use treatment, as well as the barriers unique to the rural environment, such as the lack of public transportation, that may blunt the effectiveness of peer services.

**SUMMARY BOX**

**What is already known on this topic?** Previous research has demonstrated the benefit of intervention by individuals with lived experience on cessation of substance use and preventing overdoses. Most of these interventions occurred in urban areas and inpatient settings.

**What is added by this report?** This is the first study to report the implementation of peer recovery support services for individuals in rural, Central Appalachia. Peer recovery coaches successfully engaged individuals in the emergency department and community settings and linked them to a variety of resources including housing and access to treatment. Linkage to treatment rates (63.9%) were significantly higher than have been reported in urban areas.

**What are the implications for future research?** Further elucidation of the facilitators and barriers related to implementation of peer services in the rural, Central Appalachian environment to prevent opioid overdoses is needed. The impact of peer services on linkage to treatment and other social services in the rural environment merit further investigation.

## Supplementary Information



## Figures and Tables

**Table 1:** Peer recovery support specialist engagements by referral source and linkage to treatment

Referral Source	N	% of Encounters (N=1456)	% within Referral Source
**First Responders (EMS, Police)**	**45**	**3.1**	
number that engage in substance use treatment/mental health	27		60.0
number that engage in substance use treatment/mental health for >6 months	NA		NA
number that complete substance use treatment/mental health	20		44.4
**Drug Courts-juvenile and adult**	**10**	**0.7**	
number that engage in substance use treatment/mental health	8		80.0
number that remain in substance use treatment/mental health for >6 months	5		50.0
number that complete substance use treatment/mental health	3		30.0
**Schools**	**10**	**0.7**	
number that engage in substance use treatment/mental health	10		100.0
number that remain in substance use treatment/mental health for >6 months	7		70.0
number that complete substance use treatment/mental health	4		40.0
**Social services**	**247**	**17.0**	
number that engage in substance use treatment/mental health	121		49.0
number that remain in substance use treatment/mental health for >6 months	64		25.9
number that complete substance use treatment/mental health	76		30.8
**Harm Reduction/Syringe Services Program**	**557**	**38.3**	
number that engage in substance use treatment/mental health	388		69.7
number that remain in substance use treatment/mental health for >6	96		17.2
number that complete substance use treatment/mental health	184		33.0
**Community Stakeholders**	**353**	**24.2**	
number that engage in substance use treatment/mental health	202		57.2
number that remain in substance use treatment/mental health for >6 months	45		12.7
number that complete substance use treatment/mental health	78		22.1
**Self-Referral**	**234**	**16.1**	
number that engage in substance use treatment/mental health	175		74.8
number that remain in substance use treatment/mental health for >6 months	66		28.2
number that complete substance use treatment/mental health	122		52.1

**Table 2:** Peer recovery support specialists community information sessions

	N	% of sessions (N=38)
Schools	9	23.7
Community meetings	14	36.8
Church functions	5	13.2
Others	10	26.3
