# Supplementary material for: Establishing Peer Recovery Support Services to Address the Central Appalachian Opioid Epidemic: The West Virginia Peers Enhancing Education, Recovery, and Survival (WV PEERS) Pilot Program
Source: J Appalach Health. 2021 Jul 25;3(3):36–50. doi: 10.13023/jah.0303.04 (PMC9192111; doi:10.13023/jah.0303.04)
Supplement: Supplementary file 1 [file 3.3.4Davis_AdditionalFile.pdf]

Screen

Record ID ______________________________

((INTRODUCTION SCRIPT))

Hello my name is (PRC name), I am contacting you because you expressed interest in our study after recently experiencing an opioid overdose. Before you enter the study, I would like to ask you a few question to see if you qualify. If you do qualify for the study, I would like to set-up an in person visit with you as soon as possible. The decision to participate in the study is entirely up to you and you can choose not to participate at any time. As part of this study, we will ask a few questions regarding your recent overdose experience. Then, if you are interested I can help you with educational information and with services to help prevent you from overdosing again. Also, I am here to help you find any other services you might want or need to help keep you healthy and safe. Do you have a few moments for me to ask you some question to see if you qualify for our study?

Closed to Enrollment

Today's date: ______________________________

Name of Peer Recovery Coach screening participant: ______________________________
(Please enter your first and last name.)

How did screening occur?
- ◯ Over the phone
- ◯ In person

Where was the study participant referred from?
- ◯ Dual Diagnosis Unit (DDU)
- ◯ Ruby Memorial Emergency Department
- ◯ COAT Program
- ◯ Health Right Light Program
- ◯ Friendship Room
- ◯ Bartlett House
- ◯ Valley Mental Health Services
- ◯ Morgantown Police Department
- ◯ Mon Health
- ◯ Monongalia County Health Department
- ◯ WVU Collegiate Recovery
- ◯ WVU Public Safety
- ◯ Emergency Medical Services (EMS)
- ◯ Star City Police Department
- ◯ Westover Police Department
- ◯ Self-Referred
- ◯ Other

If self-referred, how did you hear about the study? ______________________________

If other referral site, please describe: ______________________________

Is the participant at least 18 years old? ◯ Yes ◯ No

Are you currently pregnant or suspect that you are pregnant? ◯ Yes ◯ No

Do you currently have any pending criminal charges? ◯ Yes ◯ No

Do you currently have any outstanding warrants? ◯ Yes ◯ No

Are you currently on house arrest or wearing an ankle monitor? ◯ Yes ◯ No
(WILL DISQUALIFIY PARTICIPATION)

Is this person able to speak and read English? ◯ Yes ◯ No

Is the person eligible for the study? ◯ Yes ◯ No ◯ Declined Participation

If not eligible, please explain why the person is ineligible: ________________________

If the person has declined to participate, please explain: ________________________

Screening Disposition:

◯ Meets Eligibility Criteria, wants to participate
◯ Meets Eligibility Criteria, does NOT want to participate
◯ Ineligible, referred to University of Baltimore study
◯ Ineligible, Declined University of Baltimore, Referred for additional services without study enrollment

WVU IRB Approval on File
PI: Judith Feinberg
1 Medical Center Drive
Morgantown, WV 26506
Study Coordinator: Amanda Stover
304-293-5861

Closed to Enrollment

Locator

Today's date: ______________________

Participant Information

Before we begin the study visit, I am going to ask you some questions about how we can keep in contact with you for future appointments. For this section, I am going to ask you questions about yourself. Please answer as honestly and completely as possible.

Your Legal Name: ______________________
(As listed on your driver's liscense)

Street Address: ______________________
(Necessary to receive gift)

City: ______________________
(Necessary to receive gift)

State: ______________________
(please use the all capital two letter state abbreviation.)

Zipcode: ______________________

Can we send you information at this address? ◯ No ◯ Yes

Mobile/Cell Phone Number: ______________________
(must be real phone number)

When calling, do you prefer we say: ◯ WVU ◯ Staff Member Name

Can we leave a voicemail at this number? ◯ No ◯ Yes

Can we text you? ◯ No ◯ Yes

Any additional instructions or comments? ______________________

Home Phone: ______________________

When calling do you prefer we say: ◯ WVU ◯ Staff Name

Can we leave a voicemail at this number? ◯ No ◯ Yes

Instructions/Comments: ______________________

Closed to Enrollment

Alternative Forms of Communitcation

Now I am going to ask you about some other ways to contact you. This includes electronic media or messaging applications that can be used to get in touch with you by the study staff.

Email Address: ______________________

Other means of contact: __________________________________

What is the best way to leave you reminders about follow-up appointments? (check all that apply)

- ☐ Cell
- ☐ Home Phone
- ☐ Email
- ☐ Private Message on Social Network Site
- ☐ Standard Mail
- ☐ Other

Which email address? __________________________________

Which social network site? __________________________________

Other way to best contact you? __________________________________

Contact #1

Now I am going to ask you for the name of a friend or family member who can get in touch with you. This information will only be used if we are unable to contact you. No information related to the study will be revealed to this person, this person will only act as a way to contact you in case we cannot reach you based on the other information you have provided.

Closed to Enrollment

Contact (#1) Name: __________________________________

Relationship to contact #1: __________________________________

Cell phone: __________________________________

When calling do you prefer we say: ◯ WVU/MU ◯ Staff Name

Can we leave a voicemail at this number? ◯ No ◯ Yes

Instructions/Comments. __________________________________

Home phone: __________________________________

When calling this number do you prefer we say: ◯ WVU/MU ◯ Staff Name

Can we leave a message at this number (home phone)?

- ◯ No
- ◯ Yes

Comments/Instructions (home phone). __________________________________

Are there any additional ways that may be helpful to locate you? ◯ No ◯ Yes

Additional locations where we can locate you: __________________________________

Notes or additional comments:

WVU IRB Approval on File
PI: Judith Feinberg
1 Medical Center Drive
Morgantown, WV 26506
Study Coordinator: Amanda Stover
304-293-5861

Closed to Enrollment

Baseline

Participant ID ______________________
(First letter of first name + First 3 letters of last name + last 4 digits of the social security number.)

Name of Peer Recovery Coach conducting baseline: ______________________

Has participant reviewed and signed the consent document?
◯ No ◯ Yes
(If participant has NOT SIGNED CONSENT, DO NOT CONTINUE!)

Where was the visit conducted? ______________________
(Briefly describe the location (i.e. home, semi-private location like McDonald's, in the Emergency Department, etc))

Closed to Enrollment

Demographics

Today's date: ______________________

What best describes your gender?

◯ Male
◯ Female
◯ Prefer not to say
◯ Prefer to self-describe

If prefer to self-describe, please specify: ______________________

How would you describe your race or ethnicity? (Please check all that apply).

☐ White
☐ Black or African American
☐ Asian
☐ American Indian/Native Alaskan
☐ Native Hawaiian/Other Pacific Islander
☐ Other

If other race, please specify: ______________________

Do you consider yourself to be Hispanic/Latino/Latina?
◯ No ◯ Yes

What was the highest level of schooling that you have completed? (Please select only one answer)

◯ 8th grade or less
◯ Some high school
◯ High school graduate/GED
◯ Some college/Technical school/Associate's degree
◯ 4-year college degree (Bachelor's degree)
◯ Advanced degree (Master's degree, Doctorate)

If less than high school, what was the last grade you completed?

____

Overdose

I am going to ask you a few questions about overdose. An overdose is when you cannot breath and you cannot wake up without medical help.

What was the date of your most recent overdose? ________________________________

Was this overdose an accident? ○ Yes ○ No

Where did you overdose? ________________________________
(Location of where the person overdosed.)

Was this the first time you overdosed? ○ No ○ Yes ○ Don't know

How old were you when your first overdosed? ________________________________
(If s/he is unsure please encourage them to estimate as accurately as possible.)

During your LIFETIME, how many times have you overdosed on drugs?

____
(ENTER '0' IF NEVER. If s/he is unsure ask them to estimate the number as accurately as possible. Please enter a NUMBER value only.)

During the 24 hours prior to your MOST RECENT overdose, what substances did you use? (Check all that apply) (Substances include: alcoholic beverages, prescription drugs, street drugs, and over the counter drugs)

☐ Alcohol
☐ Prescription Drugs
☐ Street Drugs
☐ Over the Counter Drugs
☐ Other

If used alcohol, please specify the type of alcoholic beverage(s) you drank and how many in the 24 hours prior to your MOST RECENT overdose:

____

If used prescription drugs, please specify which prescription drugs you used, where you got them (who prescribed them or other soruce), and how much in the 24 hours prior to your MOST RECENT overdose:

____

If used street drugs, please specify the names of street drugs you used and how much in the 24 hours prior to your MOST RECENT overdose:

____

If used over the counter drugs, please specify the names of the over the counter drugs you used and how much in the 24 hours prior to your MOST RECENT overdose:

____

If any other drugs or substances were used please specify the names and how much you used in the 24 hours prior to your MOST RECENT overdose:

____

Was naloxone used to reverse your most recent overdose? ○ Yes ○ No ○ Don't Know

Closed to Enrollment

Did anyone do anything to help you when you last overdosed? ◯ Yes ◯ No

Specify what was done to help you during and after your most recent overdose: __________________________________

Did you receive any counseling or services after your overdose? ◯ Yes ◯ No

If yes, what services or type of counseling did you receive? __________________________________

If No, would you like any services or counseling? ◯ Yes ◯ No

FAMILY, WORK, & LIVING SITUATION

What is your current marital status? (Please select only one answer). ([Source: ASI])

◯ Single/Never Married
◯ Married/Living together as married
◯ Separated/Divorced
◯ Widowed
◯ Other

Please specify. other marital status: __________________________________

In the PAST MONTH, where have you been living most of the time? (DO NOT READ THE RESPONSES! Please select only ONE response)

◯ Apartment or house
◯ Residential treatment facility (mental health or substance abuse)
◯ Shelter or halfway house
◯ Institution (hospital, nursing home, jail, prison)
◯ Street/outdoors (sidewalk, park, abandoned building, car)
◯ Other

Please specify, other living situation: __________________________________

Do feel safe in the place that you CURRENTLY live?

◯ Not at all
◯ A little bit
◯ Somewhat
◯ Quite a bit
◯ Very much

Does where you currently LIVE make you want to use drugs or drink alcohol?

◯ Not at all
◯ A little bit
◯ Somewhat
◯ Quite a bit
◯ Very much

Closed to Enrollment

What is your current or usual work/employment status? (Check all that apply)
([Source: ASI])

☐ Unemployed
☐ Employed (full or part-time)
☐ Student
☐ Retired
☐ Homemaker
☐ Disabled
☐ Other

Please specify, other employment status: __________________________________

How many days in the past month were you paid for working?
(Include "under the table" work, paid sick days and vacation.) __________________________________
([Source: ASI])

Have you ever been in prison or jail at any time in your life? ◯ Yes ◯ No

When was the last time you were in prison or jail? __________________________________

Did your biological father ever have a serious problem with .....

◯ Drugs (illegal or prescription drugs)
◯ Alcohol
◯ Both alcohol & drugs
◯ Neither
◯ Unsure

Did your biological mother ever have a serious problem with .....

◯ Drugs (illegal or prescription drugs)
◯ Alcohol
◯ Both alcohol & drugs
◯ Neither
◯ Unsure

Do you have any children? ◯ Yes ◯ No

ADVERSE CHILDHOOD EXPERIENCES

These questions are about events that happened during your childhood, BEFORE you turned 18 years old. Some of the questions are sensitive. Your answers are confidential. However, if any of the questions make you uncomfortable you please let me know.

Did you live with anyone who was depressed, mentally ill, or suicidal?
([Source: WVBRFFS])

◯ No
◯ Yes
◯ Don't know/not sure

Closed to Enrollment

Did a parent, guardian or other adult in your home ever commit suicide?

◯ No
◯ Yes
◯ Don't know/not sure

Did you live with anyone who served time or was sentenced to serve time in a prison, jail or other correctional facility?
([Source: WVBRFFS])

◯ No
◯ Yes
◯ Don't know/not sure

How often did your parents, guardians or other adults in your home ever slap, hit, kick, punch or beat each other up?
([Source: WVBRFFS])

◯ Never
◯ Once
◯ More than once

How often did a parent, guardian or other adult in your home ever hit, beat, kick or physically hurt YOU in any way?

(Do NOT include spanking. [Source: WVBRFFS])

◯ Never
◯ Once
◯ More than once

How often did a parent, guardian or other adult in your home ever swear at you, insult you, or put YOU down?
(Do NOT include spanking. [Source: WVBRFFS])

◯ Never
◯ Once
◯ More than once

How often did anyone at least 5 years older than you or an adult, ever touch you sexually?
([Source: WVBRFFS])

◯ Never
◯ Once
◯ More than once

How often did anyone at least 5 years older than you or an adult, try to make you touch them sexually?
([Source: WVBRFFS])

◯ Never
◯ Once
◯ More than once

How often did anyone at least 5 years older than you or an adult, force you to have sex?
([Source: WVBRFFS])

◯ Never
◯ Once
◯ More than once

Closed to Enrollment

Before age 18 -- did a boyfriend, girlfriend or significant other ever verbally or physically abuse you?
([Source: WVBRFFS])

◯ Never
◯ Once
◯ More than once

Did your parent(s) or guardian(s) make you feel safe and protected?
([Source: WVBRFFS])

◯ No
◯ Yes
◯ Don't know/not sure

Were your parent(s) or guardian(s) ever to drunk or high to take care of you?

◯ Never
◯ Once
◯ More than once

How many times were you taken from your parent's or guardian's home and made to live somewhere else?

◯ Never
◯ Once
◯ More than once

Did you ever go without food, clothing or a place to live?

◯ Never
◯ Once
◯ More than once

INJECTION DRUG USE

Have you EVER injected drugs, even just once? ◯ Yes ◯ No

How many times have you injected drugs in the PAST MONTH? ______________________
(ENTER '0' IF NEVER. If s/he is unsure ask them to estimate the number as accurately as possible. Please enter a NUMBER value only.)

How many days in the last month did you inject opioids? ______________________
(Enter a numerical value between 0 and 30.)

Have you ever had a health or medical problem because of your injection drug use?

☐ No, Never
☐ Yes, but not in the past 3 months
☐ Yes, in the past 3 months

Have you had any of the following health problems as a result of your injection drug use?

☐ Abscess
☐ Bone infection
☐ Cellulitis
☐ Endocarditis (infection of the heart lining)
☐ Other

Specify 'other' health problem from injecting drugs:

____

Closed to Enrollment

TOBACCO, ALCOHOL, & DRUG USE

Which of the following statements best reflects your use of (drug used most) at the present time. (Select only one answer)

◯ I have stopped using
◯ I want to use more
◯ I have no desire or intention to stop using
◯ I think about not using, but have no specific plan to stop yet
◯ I have contacted a treatment agency or taken some other specific step with the intention of stopping my use of

How confident are you in your ability to be completely abstinent (clean) from alcohol and drugs in the next 30 days? ([Source: Brief Addiction Monitor])

◯ Not at all
◯ Slightly
◯ Moderately
◯ Considerably
◯ Extremely

In the past 30 days, how many days were you in any situation or with any people that might put you at an increased risk for using alcohol or drugs (for example, around risky "people, places or things")
([Source: Brief Addiction Monitor])

◯ 0
◯ 1-3
◯ 4-8
◯ 9-15
◯ 16-30

In the past 30 days, how many days did you attend self-help meetings like AA or NA to support your recovery? ([Source: Brief Addiction Monitor])

◯ 0
◯ 1-3 times
◯ 4-8 times
◯ 9-15 times
◯ 16-30 times

Have you ever, even once, used any prescription pain medication in any way a doctor did not direct you to use it?

◯ Yes ◯ No
([Include using it without a prescription of your own, using it in greater amounts, more often, or longer than you were told to take it; using it in any other way a doctor did not direct you to use it] SOURCE: NSDUH 2016)

Have you EVER used tobacco products (cigarettes, chewing tobacco, cigars, etc.) ?

◯ Yes ◯ No

How old were you when you first used a tobacco product?

____

How many days in the past month have you used tobacco products or smoked cigarettes?

____
(Days Range 0-30, Enter '0' if you have not used tobacco products in the past month)

Have you EVER drank alcoholic beverages (wine, spirits, beer, liquor, etc.)?

◯ Yes ◯ No

How old were you when you had your first drink of alcohol (more than just a sip or small taste)?

____

Closed to Enrollment

How many days in the past month have you drank alcoholic beverages? ________________________________
(Days Range 0-30, Enter '0' if you have not had alcohol in the past month)

In your life, have you EVER used cocaine or crack (blow, powder, white, rock, coke, etc.)? ◯ Yes ◯ No

How old were you when you first used cocaine or crack? ________________________________

How many days in the past month have you used cocaine or crack? ________________________________
(Days Range 0-30, Enter '0' if you have not used cocaine or crack in the past month)

In your life, have you EVER used marijuana or hashish? ◯ Yes ◯ No

How old were you when you first used marijuana or hashish? ________________________________

How many days in the past month have you used marijuana or hashish? ________________________________
(Days Range 0-30, Enter '0' if you have not used marijuana in the past month)

In your life, have you EVER used heroin? ◯ Yes ◯ No

How old were you when you FIRST USED first used heroin? ________________________________

How many days in the past month have you used heroin? ________________________________
(Days Range 0-30, Enter '0' if you have not had heroin in the past month)

In your life, have you EVER used prescription pain medication to get high? (for example, Oxycodone, hydrocodone) ◯ Yes ◯ No

How old were you when you first used prescription pain medication to get high? ________________________________

How many days in the past month did you use prescription medication to get high? ________________________________
(Days Range 0-30, Enter '0' if you have not used prescription pain pills in the past month)

In your life, have you EVER used methamphetamine or amphetamines? (for example: meth, crank, shake-and-bake, etc.) ◯ Yes ◯ No

How old were you when you first used methamphetamine or amphetamines? ________________________________

How many days in the past month have you used methamphetamines or amphetamines? ________________________________
(Days Range 0-30, Enter '0' if you have not used meth in the past month)

In your life, have you EVER used benzodiazepines (anti-anxiety) medication in a way other than prescribed by a doctor? ◯ Yes ◯ No

How old were you when you first used benzodiazepines (anti-anxiety) medications in a way other than prescribed? ________________________________

Closed to Enrollment

How many days in the past month have you misused benzodiazepines (anti-anxiety) medications?

____
(Days Range 0-30, Enter '0' if you have not used benzodiazepines or sedatives in the past month)

In your life, have you EVER used sedatives (sleep) medication in a way other than prescribed by a doctor?

◯ Yes ◯ No

How old were you when you first used sedative (sleep) medications in a way other than prescribed?

____

How many days in the past month have you misused sedative (sleep) medications?

____

In your life, have you EVER used any other drugs not mentioned?

◯ Yes ◯ No

If so, specify the name of the 'other' drug.

____

How old were you when you first used this 'other' drug?

____

Physical Health

In general, how would you rate your physical health? ([Source: PROMIS global])

◯ Excellent
◯ Very good
◯ Good
◯ Fair
◯ Poor

In general, would you say your quality of life is: ([Source: PROMIS global])

◯ Excellent
◯ Very good
◯ Good
◯ Fair
◯ Poor

Has a doctor or health professional ever told you that you have a problem with your LIVER?

◯ Yes ◯ No

If yes, which of the following liver problem(s)? (Check ALL that apply.)

☐ Hepatitis A
☐ Hepatitis B
☐ Hepatitis C
☐ Cirrhosis
☐ Other
☐ None of the above

Please specify 'other' liver problem:

____

Has a doctor or health professional ever told you that you have a sexually transmitted infection or disease (STD)?

◯ Yes ◯ No

Closed to Enrollment

If yes, has a doctor or health professional ever told you that you have any of the following: (Check ALL that apply.)

- ☐ Chlamydia
- ☐ Gonorrhea
- ☐ Syphilis
- ☐ Herpes
- ☐ HPV (genital warts)
- ☐ HIV/AIDS
- ☐ Other
- ☐ None of the above

Specify 'other' STD: ______________________

Has a doctor or health professional ever told you that you had any of these problems?

(Check ALL that apply.)

- ☐ TBI (Traumatic Brain Injury)
- ☐ Broken bones
- ☐ Stroke
- ☐ Seizures
- ☐ Other
- ☐ None of the above

Specify 'other' health problem: ______________________

HEALTH SERVICES

Do you currently have health insurance or health care coverage? ◯ Yes ◯ No

Have you EVER been treated in the emergency department (ED) for your ALCOHOL or DRUG use? ◯ Yes ◯ No
(EXCLUDE times that you were in ED for physical health problem(s))

How many times have you been treated in the EMERGENCY DEPARTMENT (ED) in the past year for your ALCOHOL or DRUG use? ______________________

Have you EVER been hospitalized (slept overnight) for your ALCOHOL or DRUG use? ◯ Yes ◯ No
(EXCLUDE times that you were in hospital for physical health problem(s))

How many nights you been hospitalized for your alcohol or drug use in the PAST YEAR? ______________________
(NIGHTS IN HOSPITAL |Response must be a number in this range: 0-365)

MENTAL HEALTH

Closedto Enrollment

In general, how would you rate your mental health, including your mood and your ability to think?
([Source: PROMIS Global4])

◯ Poor
◯ Fair
◯ Good
◯ Very good
◯ Excellent

Has a doctor or health professional ever told you that you have any of the following?

☐ Depression
☐ Bipolar Disorder
☐ Generalized Anxiety Disorder
☐ Panic Disorder
☐ Schizophrenia
☐ Post-traumatic Stress Disorder (PTSD)
☐ Ohter

If other mental health diagnosis, please specify: __________________________________

Over the past 2 weeks, how often have you been bothered by little interest or pleasure in doing things?
([Source: PHQ4])

◯ Not at all
◯ Several days
◯ More than half the days
◯ Nearly every day

Over the past 2 weeks, how often have you been bothered by feeling down, depressed or hopeless?
([Source: PHQ4])

◯ Not at all
◯ Several days
◯ More than half the days
◯ Nearly every day

In the past 7 days, I felt worthless.
([Source: PROMIS EDDEP04])

◯ Never
◯ Rarely
◯ Sometimes
◯ Usually
◯ Always

In the past 7 days, I felt helpless.
([Source: PROMIS EDDEP06])

◯ Never
◯ Rarely
◯ Sometimes
◯ Usually
◯ Always

In the past 7 days, I felt hopeless.
([Source: PROMIS EDDEP41])

◯ Never
◯ Rarely
◯ Sometimes
◯ Usually
◯ Always

Closed to Enrollment

In the past 7 days, I felt sad.
([Source: PROMIS EDDEP17])

◯ Never
◯ Rarely
◯ Sometimes
◯ Usually
◯ Always

Lately I felt cheerful.
([Source: PROMIS NQPPF22])

◯ Never
◯ Rarely
◯ Sometimes
◯ Usually
◯ Always

Mark the statement that best describes how you have been feeling for the past week, including today:
([Source: Beck Scale for Suicidal Ideation])

◯ I have a moderate to strong wish to live
◯ I have a weak wish to live
◯ I have no wish to live

Mark the statement that best describes how you have been feeling for the past week, including today:
([Source: Beck Scale for Suicidal Ideation])

◯ I would try to save my life if I found myself in a life-threatening situation
◯ I would take a chance on life or death if I found myself in a life-threatening situation
◯ I would not take the steps necessary to avoid death if I found myself in a life-threatening situation

Have you ever had thoughts of killing yourself? ◯ Yes ◯ No
([Source: SITBI-SF])

How old were you the first time you had thoughts of killing yourself ________________________

During how many separate times in your life have you had thoughts of killing yourself? (Please give your best estimate.) ________________________
([Source: SITBI-SF])

When was the LAST time?
([Source: SITBI-SF])

◯ More than one year ago
◯ In the past year
◯ In the past month
◯ In the past week
◯ In the past 24 hours

Have you ever engaged in non-suicidal self-injury? (This is bodily harm that was NOT intended to kill yourself.) ◯ Yes ◯ No
([Source: SITBI-SF])

How old were you the first time you engaged in self-harm behavior? ________________________
([Source: SITBI-SF])

How many times in your life have you engaged in non-suicidal self-harm behavior? ________________________
([Source: SITBI-SF])

Closed to Enrollment

When was the last time you engaged in non-suicidal self-harm behavior?
([Source: SITBI-SF])

◯ More than a year ago
◯ In the past year
◯ In the past month
◯ In the past week
◯ In the past 24 hours

Now I'm going to go through a list of things that people have done to harm themselves. Please let me know which of these you've done:
(PLEASE CHECK ALL THAT APPLY)

([Source: SITBI-SF])

☐ Cut or carved skin
☐ Hit yourself on purpose
☐ Pulled your hair out
☐ Gave yourself a tattoo
☐ Picked at a wound
☐ Burned your skin (i.e., with a cigarette, match or other hot object)
☐ Inserted objects under your nails or skin
☐ Bit yourself (e.g., your mouth or lip)
☐ Picked areas of your body to the point of drawing blood
☐ Scraped your skin
☐ "Erased" your skin to the point of drawing blood
☐ Other

Specify "other" method used to harm yourself: ______________________________

On average, for how long have you thought about self-harm before engaging in it?

(DO NOT read responses)
([Source: SITBI-SF])

◯ 0 seconds
◯ 1- 60 seconds
◯ 2-15 minutes
◯ 16-60 minutes
◯ less than one day
◯ 1-2 days
◯ more than two days
◯ wide range (spans > 2 responses)

Have you ever received medical treatment for non-suicidal self-harm behavior?

◯ Yes ◯ No
([Source: SITBI-SF])

Have you ever attempted to kill yourself? (check only one response)
([Source: SBQ-R])

◯ Never
◯ It was a brief passing thought
◯ I have had a plan at least once to kill myself but did not try to do it
◯ I have had a plan at least once to kill myself and really wanted to die
◯ I have attempted to kill myself, but did not want to die
◯ I have attempted to kill myself, and really hoped to die

Closed to Enrollment

How often have you thought about killing yourself?
([Source: SBQ-R])

◯ Never
◯ Rarely (1 time)
◯ Sometimes (2 times)
◯ Often (3-4 times)
◯ Very often (5 or more times)

Have you ever told someone that you were going to commit suicide or that you might do it?
([Source: SBQ-R])

◯ No
◯ Yes, at one time, but did not really want to die
◯ Yes, at one time, and really wanted to die
◯ Yes more than once, but did not want to do it
◯ Yes, more than once, and really wanted to do it

How likely is it that you will attempt suicide someday?
([Source: SBQ-R])

◯ Never
◯ No chance at all
◯ Rather unlikely
◯ Unlikely
◯ Likely
◯ Rather likely
◯ Very Likely

SOCIAL RELATIONSHIPS & ACTIVITIES

In general, how would you rate your satisfaction with your social activities and relationships?
([Source: PROMIS Global05])

◯ Poor
◯ Fair
◯ Good
◯ Very good
◯ Excellent

I have someone who will listen to me when I need to talk.
([Source: PROMIS FSE31053x2])

◯ Never
◯ Rarely
◯ Sometimes
◯ Usually
◯ Always

I have someone who understands my problems.
([Source: PROMIS FSE31069x2])

◯ Never
◯ Rarely
◯ Sometimes
◯ Usually
◯ Always

Closed to Enrollment

Substance Use Treatment

Have you ever received treatment or counseling for your use of alcohol or any drug, not counting cigarettes?

◯ No ◯ Yes
(SOURCE: NSDUH 2016)

How many times in your life have you received inpatient help (stayed somewehre overnight or for multiple days) for your use of drugs?

____

In the past 90 days, have you received any type of professional help for your drug use (where you did not stay overnight in a facility)?

◯ Yes ◯ No

How old you were you the first time you received drug treatment?

____

During the past year, have you received treatment for your use of alcohol or drugs at any of the following? (Check ALL that apply)
(source: modified NSDUH, 2016)

☐ A drug or alcohol rehab facility as an outpatient
☐ A drug or alcohol rehab facility as an inpatient
☐ A private doctor's office
☐ A prison or jail
☐ Self-help group (such as Alcoholic/Narcotics Anonymous)
☐ Home, family, friends
☐ School, college
☐ Church/religious/spiritual organization
☐ Mental Health Rehab/intervention as an inpatient
☐ Mental Health Rehab/intervention as an outpatient
☐ Counselor, therapist, psychologist, psychiatrist
☐ Court-mandated/sponsored program
☐ Alcohol/drug class or education
☐ Methadone clinic/program
☐ Buprenorphine clinic/progam
☐ Emergency Department
☐ Other

Other, please specify:

____

Miscellaneous Notes:

Closed to Enrollment

WVU IRB Approval on File
PI: Judith Feinberg
1 Medical Center Drive
Morgantown, WV 26506
Study Coordinator: Amanda Stover
304-293-5861

Referrals to Services

You are important. I want to talk to you about some ways that I can help you. I would like to know what I can do to help keep you healthy and safe.

Today's Date: ______________________

Interaction was completed: ◯ By Phone ◯ In Person

ADDICTION SERVICES

Participant was connected with the following services for Addiction: (Please select all that apply).

- ☐ Medication Assisted Treatment (Buprenorphine)
- ☐ Medication Assisted Treatment (Methadone)
- ☐ Medication Assisted Treatment (Naltrexone)
- ☐ Therapy
- ☐ Support Groups
- ☐ Recovery Housing
- ☐ Drop-in Center
- ☐ Other

Where was participant referred for Medication Assisted Treatment? ______________________

Where was participant referred for thearpy? ______________________

What support group/groups was the participant referred to? ______________________

Where was the participant referred for recovery housing? ______________________

What drop in center was the participant referred to? ______________________

What other addiction services was the participant referred to? ______________________

HARM REDUCTION SERVICES

Participant was connected with the following services for Harm Recuction: (Please select all that apply).

- ☐ Safe Injection Practices/Education
- ☐ Clean Injection Equipment
- ☐ Overdose Prevention Education
- ☐ Naloxone
- ☐ Other

Where was the participant referred for harm reduction services? ______________________

Where was the participant referred for naloxone? ______________________

Closed to Enrollment

What other harm reduction services was that participant referred to? __________________________________

INFECTIOUS DISEASE SERVICES

Participant was connected with the following services for Infectious Diseases: (Please select all that apply).

- ☐ Hepatitis C Prevention Education
- ☐ Hepatitis C Testing
- ☐ Hepatitis C Treatment
- ☐ HIV/AIDS Prevention Education
- ☐ HIV/AIDS Testing
- ☐ HIV/AIDS Treatment
- ☐ STI/STD Testing
- ☐ STI/STD Treatment
- ☐ Skin or Soft Tissue Infection Treatment
- ☐ Other

Where was the participant referred for Hepatitis C testing? __________________________________

Where was the participant referred for Hepatitis C treatment? __________________________________

Where was the participant referred for HIV/AIDS testing? __________________________________

Where was the participant referred for HIV/AIDS treatment? __________________________________

Where was the participant referred for STI/STD testing? __________________________________

Where was the participant referred for STI/STD treatment? __________________________________

Where was the participant referred for skin/soft tissue infection treatment? __________________________________

Sepcify other infection disease treatment referal: __________________________________

Where was the participant referred? __________________________________

Closed to Enrollment

MENTAL HEALTH SERVICES

Participant was connected with the following services for Mental Health: (Please select all that apply).

- ☐ Therapy
- ☐ Medication
- ☐ Suicide Prevention/Assessment
- ☐ Evaluation
- ☐ Inpatient Services
- ☐ Crisis Management
- ☐ Other

Where was the participant referred for therapy? __________________________________

Where was the participant referred for medication? __________________________________

Where was the participant referred for suicide prevention services/assessment? __________________________________

Where was the participant referred for mental health evaluation? __________________________________

Where was the participant referred for inpatient mental health services? __________________________________

Where was the participant referred for additional crisis management? __________________________________

Where was the participant referred for other mental health services? __________________________________

PRIMARY CARE SERVICES

Participant was connected with the following services for Primary Care: (Please select all that apply).

☐ General Healthcare
☐ Chronic Disease Management
☐ Vaccinations
☐ Acute Illness
☐ Women's Health
☐ Other

Where was the participant referred for general healthcare services? __________________________________

Where was the participant referred for chronic disease management? __________________________________

Where was the participant referred for vaccinations? __________________________________

What vaccination(s) was the participant interested in receiving? __________________________________

Where was the participant referred for actue illness? __________________________________

Where was the participant referred for women's healthcare? __________________________________

What other primary care services was the participant referred to? __________________________________

Closed to Enrollment

SOCIAL SERVICES

Participant was connected with the following services for Social Services: (Please select all that apply).

☐ Legal Services
☐ Child Care
☐ Insurance
☐ Employment
☐ Transportation
☐ Housing
☐ Clothing
☐ Eye Care
☐ Dental
☐ Food
☐ Emergency Assistance
☐ Abuse Support
☐ Family Services
☐ Schooling/Professional Training
☐ Other

Where was the participant referred for legal services? ______________________________

Where was the participant referred for child care/services? ______________________________

Where was the participant referred for insurance? ______________________________

Where was the participant referred for employment? ______________________________

Where was the participant referred for housing? ______________________________

Where was the participant referred for clothing? ______________________________

Where was the participant referred for eye care? ______________________________

Where was the participant referred for dental? ______________________________

Where was the participant referred for food assistance? ______________________________

Where was the participant referred for emergency assistance? ______________________________

Where was the participant referred for abuse suport? ______________________________

Where was the participant referred for family services? ______________________________

Where was the participant referred for schooling or professional training? ______________________________

Where was the participant referred for other social services? ______________________________

WVU IRB Approval on File
PI: Judith Feinberg
1 Medical Center Drive
Morgantown, WV 26506
Study Coordinator: Amanda Stover
304-293-5861

Closed to Enrollment

Tracking 1-Month Followup Interviews

Date of first follow-up phone call ________________________________

Results of first follow-up call
○ No response & left voicemail message
○ Phone number out of service
○ No response & no voicemail (mail box full or not set-up)
○ Follow-up completed
○ Other

Specify other result of first follow-up phone call attempt ________________________________

Date of first email attempt ________________________________

Result of first email attempt ________________________________

SECOND ATTEMPT TO CONTACT FOR FOLLOW-UP

Date of second follow-up phone call ________________________________

Results of second follow-up call
○ No response & left voicemail message
○ Phone number out of service
○ No response & no voicemail (mail box full or not set-up)
○ Follow-up completed
○ Other

Specify other result of second follow-up call attempt ________________________________

Date of second email attempt ________________________________

Result of second email attempt ________________________________

THIRD ATTEMPT TO FOLLOW-UP

Date of third follow-up phone call ________________________________

Result of third follow-up phone call
○ No response & left voicemail message
○ Phone number out of service
○ No response & no voicemail (mail box full or not set-up)
○ Follow-up completed
○ Other

Specify other result of third follow-up attempt phone call ________________________________

Date of third email attempt ________________________________

Result of third email attempt ________________________________

Closed to Enrollment

FOURTH ATTEMPT TO FOLLOW-UP

Date of fourth follow-up phone call ________________________________

Result of fourth follow-up phone call
- ◯ No response & left voicemail message
- ◯ Phone number out of service
- ◯ No response & no voicemail (mail box full or not set-up)
- ◯ Follow-up completed
- ◯ Other

Specify other result of fourth follow-up attempt phone call ________________________________

Date of fourth email attempt ________________________________

Result of fourth email attempt ________________________________

FIFTH ATTEMPT FOLLOW-UP

Date of fifth follow-up phone call ________________________________

Result of fifth follow-up phone call
- ◯ No response & left voicemail message
- ◯ Phone number out of service
- ◯ No response & no voicemail (mail box full or not set-up)
- ◯ Follow-up completed
- ◯ Other

Specify other result of fifth follow-up phone call attempt ________________________________

Date of fifth email attempt ________________________________

Result of fifth email attempt ________________________________

SIXTH ATTEMPT TO FOLLOW-UP

Date of sixth follow-up phone call ________________________________

Result of sixth follow-up phone call
- ◯ No response & left voicemail message
- ◯ Phone number out of service
- ◯ No response & no voicemail (mail box full or not set-up)
- ◯ Follow-up completed
- ◯ Other

Specify other result of sixth follow-up phone call attempt ________________________________

Date of sixth email attempt ________________________________

Result of sixth email attempt ________________________________

Closed to Enrollment

FOLLOW UP DISPOSITION STATUS

Discontinued study participation due to:

◯ Failure to respond to PRC contact
◯ Voluntarily withdrew
◯ Death
◯ Arrest
◯ Pregnancy
◯ Other

Please specify other reason for discontinuation: ______________________

Follow-up notes:

WVU IRB Approval on File
PI: Judith Feinberg
1 Medical Center Drive
Morgantown, WV 26506
Study Coordinator: Amanda Stover
304-293-5861

Closed to Enrollment

Follow-Up (1 Month)

Participant ID ______________________________
(First letter of first name + First 3 letters of last name + last 4 digits of the social security number.)

Name of Peer Recovery Coach: ______________________________

Where was this visit conducted? ______________________________

Today's date: ______________________________

Overdose

Have you experienced an overdose in the past 30 days? ◯ Yes ◯ No

What was the date of your most recent overdose? ______________________________

Was this overdose an accident? ◯ Yes ◯ No

Where did you overdose? ______________________________
(Location of where the person overdosed.)

During the 24 hours prior to your MOST RECENT overdose, what substances did you use? (Check all that apply) (Substances include: alcoholic beverages, prescription drugs, street drugs, and over the counter drugs)

☐ Alcohol
☐ Prescription Drugs
☐ Street Drugs
☐ Over the Counter Drugs
☐ Other

If used alcohol, please specify the type of alcoholic beverage(s) you drank and how many in the 24 hours prior to your MOST RECENT overdose:

____

If used prescription drugs, please specify which prescription drugs you used and how much in the 24 hours prior to your MOST RECENT overdose:

____

If used street drugs, please specify the names of street drugs you used and how much in the 24 hours prior to your MOST RECENT overdose:

____

If used over the counter drugs, please specify the names of the over the counter drugs you used and how much in the 24 hours prior to your MOST RECENT overdose:

____

If any other drugs or substances were used please specify the names and how much you used in the 24 hours prior to your MOST RECENT overdose:

____

Closed to Enrollment

Was naloxone used to reverse your most recent overdose? ◯ Yes ◯ No ◯ Don't Know

Did anyone do anything to help you when you last overdosed? ◯ Yes ◯ No

Specify what was done to help you during and after your most recent overdose: ______________________

Did you receive any counseling or services after your overdose? ◯ Yes ◯ No

If yes, what services or type of counseling did you receive? ______________________

If No, would you like any services or counseling? ◯ Yes ◯ No

FAMILY, WORK, & LIVING SITUATION

In the PAST MONTH, where have you been living most of the time? (Please select only one response) (DO NOT READ THE RESPONSES!)

◯ Apartment or house
◯ Residential treatment facility (mental health or substance abuse)
◯ Shelter or halfway house
◯ Institution (hospital, nursing home, jail, prison)
◯ Street/outdoors (sidewalk, park, abandoned building, car)
◯ Other

If other living situation than listed above, please specify: ______________________

Do feel safe in the place that you CURRENTLY live?

◯ Not at all
◯ A little bit
◯ Somewhat
◯ Quite a bit
◯ Very much

Does where you currently LIVE make you want to use drugs or drink alcohol?

◯ Not at all
◯ A little bit
◯ Somewhat
◯ Quite a bit
◯ Very much

What is your current or usual work/employment status? (Check all that apply) ([Source: ASI, 5th Ed. 1992])

☐ Unemployed
☐ Employed (full or part-time)
☐ Student
☐ Retired
☐ Homemaker
☐ Disabled
☐ Other

If other employment status, please specify: ______________________

Closed to Enrollment

How many days in the past month were you paid for working?
(Include "under the table" work, paid sick days and vacation.)

____
([Source: ASI, 5th Ed. 1992])

INJECTION DRUG USE

How many times have you injected drugs in the PAST MONTH?

____

How many days in the last month did you inject opioids?

____
(Enter a numerical value between 0 and 30.)

In the past 3 months, have you had any of the following as a result of your injection drug use?

☐ Abscess
☐ Bone infection
☐ Cellulitis
☐ Endocarditis (infection of the heart lining)
☐ Other

Specify 'other' health problem from injecting drugs:

____

TOBACCO, ALCOHOL, & DRUG USE

Which of the following statements best reflects your use of (drug used most) at the present time. (Select only one answer)

◯ I have stopped using
◯ I want to use more
◯ I have no desire or intention to stop using
◯ I think about not using, but have no specific plan to stop yet
◯ I have contacted a treatment agency or taken some other specific step with the intention of stopping my use of

How confident are you in your ability to be completely abstinent (clean) from alcohol and drugs in the next 30 days?
([Source: Brief Addiction Monitor])

◯ Not at all
◯ Slightly
◯ Moderately
◯ Considerably
◯ Extremely

In the past 30 days, how many days were you in any situation or with any people that might put you at an increased risk for using alcohol or drugs (for example, around risky "people, places or things")
([Source: Brief Addiction Monitor])

◯ 0
◯ 1-3
◯ 4-8
◯ 9-15
◯ 16-30

Closed to Enrollment

In the past 30 days, how many days did you attend self-help meetings like AA or NA to support your recovery? ([Source: Brief Addiction Monitor])

◯ 0
◯ 1-3 times
◯ 4-8 times
◯ 9-15 times
◯ 16-30 times

How many days in the past month have you used tobacco products or smoked cigarettes? ________________________________ (Days Range 0-30, Enter '0' if you have not used tobacco products in the past month)

How many days in the past month have you drank alcoholic beverages? ________________________________ (Days Range 0-30, Enter '0' if you have not had alcohol in the past month)

How many days in the past month have you used cocaine or crack? ________________________________ (Days Range 0-30, Enter '0' if you have not used cocaine or crack in the past month)

How many days in the past month have you used marijuana or hashish? ________________________________ (Days Range 0-30, Enter '0' if you have not used marijuana in the past month)

How many days in the past month have you used heroin? ________________________________ (Days Range 0-30, Enter '0' if you have not had heroin in the past month)

How many days in the past month did you use prescription medication to get high? ________________________________ (Days Range 0-30, Enter '0' if you have not used prescription pain pills in the past month)

How many days in the past month have you used methamphetamines or amphetamines? ________________________________ (Days Range 0-30, Enter '0' if you have not used meth in the past month)

How many days in the past month have you misused benzodiazepines (anti-anxiety) medications? ________________________________ (Days Range 0-30, Enter '0' if you have not used benzodiazepines or sedatives in the past month)

How many days in the past month have you misused sedative (sleep) medications? ________________________________

In the past month have you misused any other drugs not mentioned? ◯ Yes ◯ No

If yes, please specify the other drug or drugs you've misused. ________________________________

Closed to Enrollment

Physical Health

In general, how would you rate your physical health? ([Source: PROMIS global])

◯ Excellent
◯ Very good
◯ Good
◯ Fair
◯ Poor

In general, would you say your quality of life is:
([Source: PROMIS global])

◯ Excellent
◯ Very good
◯ Good
◯ Fair
◯ Poor

In the past 30 days, has a doctor or health professional told you that you have a problem with your LIVER? ◯ Yes ◯ No

If yes, which of the following liver problems or problem do you have?
(Check ALL that apply.)

☐ Hepatitis A
☐ Hepatitis B
☐ Hepatitis C
☐ Cirrhosis
☐ Other
☐ None of the above

Please specify 'other' liver problem: ______________________________

In the past 30 days, has a doctor or health professional told you that you have a sexually transmitted infection or disease? ◯ Yes ◯ No

If yes, has a doctor or health professional told you that you have any of the following:
(Check ALL that apply.)

☐ Chlamydia
☐ Gonorrhea
☐ Syphilis
☐ Herpes
☐ HPV (genital warts)
☐ HIV/AIDS
☐ Other
☐ None of the above

Specify 'other' STD: ______________________________

In the past 30 days, has a doctor or health professional told you that you have any of these problems?

(Check ALL that apply.)

☐ TBI (Traumatic Brain Injury)
☐ Broken bones
☐ Stroke
☐ Seizures
☐ Other
☐ None of the above

Specify 'other' health problem: ______________________________

Closed to Enrollment

HEALTH SERVICES

Do you currently have health insurance or health care coverage? ◯ Yes ◯ No

In the past 30 days have you been treated in the emergency department (ED) for your ALCOHOL or DRUG use? ◯ Yes ◯ No
(EXCLUDE times that you were in ED for physical health problem(s))

How many times have you been treated in the EMERGENCY DEPARTMENT (ED) in the past 90 days for your ALCOHOL or DRUG use? ________________________________

In the past 30 days, have you been hospitalized (slept overnight) for your ALCOHOL or DRUG use? ◯ Yes ◯ No
(EXCLUDE times that you were in hospital for physical health problem(s))

In the past 30 days, how many nights you been hospitalized for your alcohol or drug use? ________________________________
(NIGHTS IN HOSPITAL |Response must be a number in this range: 0-365)

MENTAL HEALTH

In general, how would you rate your mental health, including your mood and your ability to think? ([Source: PROMIS Global4])

◯ Poor
◯ Fair
◯ Good
◯ Very good
◯ Excellent

In the past 30 days, has a doctor or health professional told you that you have any of the following?

☐ Depression
☐ Bipolar Disorder
☐ Generalized Anxiety Disorder
☐ Panic Disorder
☐ Schizophrenia
☐ Post-traumatic Stress Disorder (PTSD)
☐ Ohter

If other mental health diagnosis, please specify: ________________________________

Over the past 2 weeks, how often have you been bothered by little interest or pleasure in doing things? ([Source: PHQ4])

◯ Not at all
◯ Several days
◯ More than half the days
◯ Nearly every day

Over the past 2 weeks, how often have you been bothered by feeling down, depressed or hopeless? ([Source: PHQ4])

◯ Not at all
◯ Several days
◯ More than half the days
◯ Nearly every day

Closed to Enrollment

In the past 7 days, I felt worthless.
([Source: PROMIS EDDEP04])

◯ Never
◯ Rarely
◯ Sometimes
◯ Usually
◯ Always

In the past 7 days, I felt helpless.
([Source: PROMIS EDDEP06])

◯ Never
◯ Rarely
◯ Sometimes
◯ Usually
◯ Always

In the past 7 days, I felt hopeless.
([Source: PROMIS EDDEP41])

◯ Never
◯ Rarely
◯ Sometimes
◯ Usually
◯ Always

In the past 7 days, I felt sad.
([Source: PROMIS EDDEP17])

◯ Never
◯ Rarely
◯ Sometimes
◯ Usually
◯ Always

Lately I felt cheerful.
([Source: PROMIS NQPPF22])

◯ Never
◯ Rarely
◯ Sometimes
◯ Usually
◯ Always

Mark the statement that best describes how you have been feeling for the past week, including today:
([Source: Beck Scale for Suicidal Ideation])

◯ I have a moderate to strong wish to live
◯ I have a weak wish to live
◯ I have no wish to live

Mark the statement that best describes how you have been feeling for the past week, including today:
([Source: Beck Scale for Suicidal Ideation])

◯ I would try to save my life if I found myself in a life-threatening situation
◯ I would take a chance on life or death if I found myself in a life-threatening situation
◯ I would not take the steps necessary to avoid death if I found myself in a life-threatening situation

In the past 30 days have you had thoughts of killing yourself?

◯ Yes ◯ No
([Source: SITBI-SF])

Closed to Enrollment

In the past 30 days, how many separate times did you have thoughts of killing yourself? (Please give your best estimate.)

____
([Source: SITBI-SF, modified to reflect last 30 days])

When was the LAST time?
([Source: SITBI-SF, modified to reflect time period])

◯ In the past month
◯ In the past week
◯ In the past 24 hours

Have you ever engaged in non-suicidal self-injury? (This is bodily harm that was NOT intended to kill yourself.)

◯ Yes ◯ No
([Source: SITBI-SF])

In the past 30 days, how many times have you engaged in non-suicidal self-harm behavior?

____
([Source: SITBI-SF, modified to reflect time period])

When was the last time you engaged in non-suicidal self-harm behavior?
([Source: SITBI-SF, modified to reflect time period])

◯ In the past month
◯ In the past week
◯ In the past 24 hours

Now I'm going to go through a list of things that people have done to harm themselves. Please let me know which of these you've done, in the past 30 DAYS:
(PLEASE CHECK ALL THAT APPLY)

([Source: SITBI-SF, modified to reflect time period])

☐ cut or carved skin
☐ hit yourself on purpose
☐ pulled your hair out
☐ gave yourself a tattoo
☐ picked at a wound
☐ burned your skin (i.e., with a cigarette, match or other hot object)
☐ inserted objects under your nails or skin
☐ bit yourself (e.g., your mouth or lip)
☐ picked areas of your body to the point of drawing blood
☐ scraped your skin
☐ "erased" your skin to the point of drawing blood
☐ other

On average, for how long have you thought about self-harm before engaging in it?

(DO NOT read responses)
([Source: SITBI-SF])

◯ 0 seconds
◯ 1- 60 seconds
◯ 2-15 minutes
◯ 16-60 minutes
◯ less than one day
◯ 1-2 days
◯ more than two days
◯ wide range (spans > 2 responses)

In the past 30 days, have you received medical treatment for non-suicidal self-harm behavior?

◯ Yes ◯ No
([Source: SITBI-SF, modified to reflect time period])

Closed to Enrollment

In the past 30 days, have you attempted to kill yourself? (check only one response)
([Source: SBQ-R, modified to reflect time period])

◯ Never
◯ It was a brief passing thought
◯ I have had a plan at least once to kill myself but did not try to do it
◯ I have had a plan at least once to kill myself and really wanted to die
◯ I have attempted to kill myself, but did not want to die
◯ I have attempted to kill myself, and really hoped to die

In the past 30 days, how often have you thought about killing yourself?
([Source: SBQ-R, modified to reflect time period])

◯ Never
◯ Rarely (1 time)
◯ Sometimes (2 times)
◯ Often (3-4 times)
◯ Very often (5 or more times)

In the past 30 days, have you ever told someone that you were going to commit suicide or that you might do it?
([Source: SBQ-R, modified to reflect time period])

◯ No
◯ Yes, at one time, but did not really want to die
◯ Yes, at one time, and really wanted to die
◯ Yes more than once, but did not want to do it
◯ Yes, more than once, and really wanted to do it

How likely is it that you will attempt suicide someday?
([Source: SBQ-R])

◯ Never
◯ No chance at all
◯ Rather unlikely
◯ Unlikely
◯ Likely
◯ Rather likely
◯ Very Likely

Closed to Enrollment

SOCIAL RELATIONSHIPS & ACTIVITIES

In general, how would you rate your satisfaction with your social activities and relationships?
([Source: PROMIS Global05])

◯ Poor
◯ Fair
◯ Good
◯ Very good
◯ Excellent

I have someone who will listen to me when I need to talk.
([Source: PROMIS FSE31053x2])

◯ Never
◯ Rarely
◯ Sometimes
◯ Usually
◯ Always

I have someone who understands my problems.
([Source: PROMIS FSE31069x2])

◯ Never
◯ Rarely
◯ Sometimes
◯ Usually
◯ Always

Substance Use Treatment

In the past 30 days, have you ever received treatment or counseling for your use of alcohol or any drug, not counting cigarettes?

◯ No ◯ Yes
(SOURCE: NSDUH 2016)

During the past 30 days, have you received treatment for your use of alcohol or drugs at any of the following?
(source: modified NSDUH, 2016)

☐ A drug or alcohol rehab facility as an outpatient
☐ A drug or alcohol rehab facility as an inpatient
☐ A private doctor's office
☐ A prison or jail
☐ Self-help group (such as Alcoholic/Narcotics Anonymous)
☐ Home, family, friends
☐ School, college
☐ Church/religious/spiritual organization
☐ Mental Health Rehab/intervention as an inpatient
☐ Mental Health Rehab/intervention as an outpatient
☐ Counselor, therapist, psychologist, psychiatrist
☐ Court-mandated/sponsored program
☐ Alcohol/drug class or education
☐ Methadone clinic/program
☐ Buprenorphine clinic/progam
☐ Emergency Department
☐ Other

Other, please specify: ______________________________

Miscellaneous Notes:

Closed to Enrollment

WVU IRB Approval on File
PI: Judith Feinberg
1 Medical Center Drive
Morgantown, WV 26506
Study Coordinator: Amanda Stover
304-293-5861

Referrals to Services (1 Month FU)

You are important. I want to talk to you about some ways that I can help you. I would like to know what I can do to help keep you healthy and safe.

Today's Date: ______________________

Interaction was completed: ◯ By Phone ◯ In Person

ADDICTION SERVICES

Participant was connected with the following services for Addiction: (Please select all that apply).

- ☐ Medication Assisted Treatment (Buprenorphine)
- ☐ Medication Assisted Treatment (Methadone)
- ☐ Medication Assisted Treatment (Naltrexone)
- ☐ Therapy
- ☐ Support Groups
- ☐ Recovery Housing
- ☐ Drop-in Center
- ☐ Other

Where was participant referred for Medication Assisted Treatment? ______________________

Where was participant referred for thearpy? ______________________

What support group/groups was the participant referred to? ______________________

Where was the participant referred for recovery housing? ______________________

What drop in center was the participant referred to? ______________________

What other addiction services was the participant referred to? ______________________

HARM REDUCTION SERVICES

Participant was connected with the following services for Harm Recuction: (Please select all that apply).

- ☐ Safe Injection Practices/Education
- ☐ Clean Injection Equipment
- ☐ Overdose Prevention Education
- ☐ Naloxone
- ☐ Other

Where was the participant referred for harm reduction services? ______________________

Where was the participant referred for naloxone? ______________________

Closed to Enrollment

What other harm reduction services was that participant referred to? __________________________________

INFECTIOUS DISEASE SERVICES

Participant was connected with the following services for Infectious Diseases: (Please select all that apply).

☐ Hepatitis C Prevention Education
☐ Hepatitis C Testing
☐ Hepatitis C Treatment
☐ HIV/AIDS Prevention Education
☐ HIV/AIDS Testing
☐ HIV/AIDS Treatment
☐ STI/STD Testing
☐ STI/STD Treatment
☐ Skin or Soft Tissue Infection Treatment
☐ Other

Where was the participant referred for Hepatitis C testing? __________________________________

Where was the participant referred for Hepatitis C treatment? __________________________________

Where was the participant referred for HIV/AIDS testing? __________________________________

Where was the participant referred for HIV/AIDS treatment? __________________________________

Where was the participant referred for STI/STD testing? __________________________________

Where was the participant referred for STI/STD treatment? __________________________________

Where was the participant referred for skin/soft tissue infection treatment? __________________________________

Sepcify other infection disease treatment referal: __________________________________

Where was the participant referred? __________________________________

Closed to Enrollment

MENTAL HEALTH SERVICES

Participant was connected with the following services for Mental Health: (Please select all that apply).

☐ Therapy
☐ Medication
☐ Suicide Prevention/Assessment
☐ Evaluation
☐ Inpatient Services
☐ Crisis Management
☐ Other

Where was the participant referred for therapy? __________________________________

Where was the participant referred for medication? __________________________________

Where was the participant referred for suicide prevention services/assessment? ____________________

Where was the participant referred for mental health evaluation? ____________________

Where was the participant referred for inpatient mental health services? ____________________

Where was the participant referred for additional crisis management? ____________________

Where was the participant referred for other mental health services? ____________________

PRIMARY CARE SERVICES

Participant was connected with the following services for Primary Care: (Please select all that apply).

☐ General Healthcare
☐ Chronic Disease Management
☐ Vaccinations
☐ Acute Illness
☐ Women's Health
☐ Other

Where was the participant referred for general healthcare services? ____________________

Where was the participant referred for chronic disease management? ____________________

Where was the participant referred for vaccinations? ____________________

What vaccination(s) was the participant interested in receiving? ____________________

Where was the participant referred for actue illness? ____________________

Where was the participant referred for women's healthcare? ____________________

What other primary care services was the participant referred to? ____________________

SOCIAL SERVICES

Closed to Enrollment

Participant was connected with the following services for Social Services: (Please select all that apply).

☐ Legal Services
☐ Child Care
☐ Insurance
☐ Employment
☐ Transportation
☐ Housing
☐ Clothing
☐ Eye Care
☐ Dental
☐ Food
☐ Emergency Assistance
☐ Abuse Support
☐ Family Services
☐ Schooling/Professional Training
☐ Other

Where was the participant referred for legal services? ________________________________

Where was the participant referred for child care/services? ________________________________

Where was the participant referred for insurance? ________________________________

Where was the participant referred for employment? ________________________________

Where was the participant referred for housing? ________________________________

Where was the participant referred for clothing? ________________________________

Where was the participant referred for eye care? ________________________________

Where was the participant referred for dental? ________________________________

Where was the participant referred for food assistance? ________________________________

Where was the participant referred for emergency assistance? ________________________________

Where was the participant referred for abuse suport? ________________________________

Where was the participant referred for family services? ________________________________

Where was the participant referred for schooling or professional training? ________________________________

Where was the participant referred for other social services? ________________________________

WVU IRB Approval on File
PI: Judith Feinberg
1 Medical Center Drive
Morgantown, WV 26506
Study Coordinator: Amanda Stover
304-293-5861

Closed to Enrollment

Tracking 3-Month Followup Interviews

Date of first follow-up phone call ________________

Results of first follow-up call
- ◯ No response & left voicemail message
- ◯ Phone number out of service
- ◯ No response & no voicemail (mail box full or not set-up)
- ◯ Follow-up completed
- ◯ Other

Specify other result of first follow-up phone call attempt ________________

Date of first email attempt ________________

Result of first email attempt ________________

SECOND ATTEMPT TO CONTACT FOR FOLLOW-UP

Date of second follow-up phone call ________________

Results of second follow-up call
- ◯ No response & left voicemail message
- ◯ Phone number out of service
- ◯ No response & no voicemail (mail box full or not set-up)
- ◯ Follow-up completed
- ◯ Other

Specify other result of second follow-up call attempt ________________

Date of second email attempt ________________

Result of second email attempt ________________

THIRD ATTEMPT TO FOLLOW-UP

Date of third follow-up phone call ________________

Result of third follow-up phone call
- ◯ No response & left voicemail message
- ◯ Phone number out of service
- ◯ No response & no voicemail (mail box full or not set-up)
- ◯ Follow-up completed
- ◯ Other

Specify other result of third follow-up attempt phone call ________________

Date of third email attempt ________________

Result of third email attempt ________________

Closed to Enrollment

FOURTH ATTEMPT TO FOLLOW-UP

Date of fourth follow-up phone call ______________________

Result of fourth follow-up phone call
- ◯ No response & left voicemail message
- ◯ Phone number out of service
- ◯ No response & no voicemail (mail box full or not set-up)
- ◯ Follow-up completed
- ◯ Other

Specify other result of fourth follow-up attempt phone call ______________________

Date of fourth email attempt ______________________

Result of fourth email attempt ______________________

FIFTH ATTEMPT FOLLOW-UP

Date of fifth follow-up phone call ______________________

Result of fifth follow-up phone call
- ◯ No response & left voicemail message
- ◯ Phone number out of service
- ◯ No response & no voicemail (mail box full or not set-up)
- ◯ Follow-up completed
- ◯ Other

Specify other result of fifth follow-up phone call attempt ______________________

Date of fifth email attempt ______________________

Result of fifth email attempt ______________________

SIXTH ATTEMPT TO FOLLOW-UP

Date of sixth follow-up phone call ______________________

Result of sixth follow-up phone call
- ◯ No response & left voicemail message
- ◯ Phone number out of service
- ◯ No response & no voicemail (mail box full or not set-up)
- ◯ Follow-up completed
- ◯ Other

Specify other result of sixth follow-up phone call attempt ______________________

Date of sixth email attempt ______________________

Result of sixth email attempt ______________________

Closed to Enrollment

FOLLOW UP DISPOSITION STATUS

Discontinued study participation due to:

◯ Failure to respond to PRC contact
◯ Voluntarily withdrew
◯ Death
◯ Arrest
◯ Pregnancy
◯ Other

Please specify other reason for discontinuation: ______________________________

Follow-up notes:

WVU IRB Approval on File
PI: Judith Feinberg
1 Medical Center Drive
Morgantown, WV 26506
Study Coordinator: Amanda Stover
304-293-5861

Closed to Enrollment

Follow-Up (3 Month)

Participant ID ______________________
(First letter of first name + First 3 letters of last name + last 4 digits of the social security number.)

Name of Peer Recovery Coach: ______________________

Where was this visit conducted? ______________________

Today's date: ______________________

Overdose

Have you experienced an overdose in the past 90 days? ○ Yes ○ No

What was the date of your most recent overdose? ______________________

Was this overdose an accident? ○ Yes ○ No

Where did you overdose? ______________________
(Location of where the person overdosed.)

During the 24 hours prior to your MOST RECENT overdose, what substances did you use? (Check all that apply)
(Substances include: alcoholic beverages, prescription drugs, street drugs, and over the counter drugs)

☐ Alcohol
☐ Prescription Drugs
☐ Street Drugs
☐ Over the Counter Drugs
☐ Other

If used alcohol, please specify the type of alcoholic beverage(s) you drank and how many in the 24 hours prior to your MOST RECENT overdose:

____

If used prescription drugs, please specify which prescription drugs you used and how much in the 24 hours prior to your MOST RECENT overdose:

____

If used street drugs, please specify the names of street drugs you used and how much in the 24 hours prior to your MOST RECENT overdose:

____

If used over the counter drugs, please specify the names of the over the counter drugs you used and how much in the 24 hours prior to your MOST RECENT overdose:

____

If any other drugs or substances were used please specify the names and how much you used in the 24 hours prior to your MOST RECENT overdose:

____

Closed to Enrollment

Was naloxone used to reverse your most recent overdose? ◯ Yes ◯ No ◯ Don't Know

Did anyone do anything to help you when you last overdosed? ◯ Yes ◯ No

Specify what was done to help you during and after your most recent overdose: __________________________________

Did you receive any counseling or services after your overdose? ◯ Yes ◯ No

If yes, what services or type of counseling did you receive? __________________________________

If No, would you like any services or counseling? ◯ Yes ◯ No

FAMILY, WORK, & LIVING SITUATION

In the PAST MONTH, where have you been living most of the time? (Please select only one response) (DO NOT READ THE RESPONSES!)

◯ Apartment or house
◯ Residential treatment facility (mental health or substance abuse)
◯ Shelter or halfway house
◯ Institution (hospital, nursing home, jail, prison)
◯ Street/outdoors (sidewalk, park, abandoned building, car)
◯ Other

If other living situation than listed above, please specify: __________________________________

Do feel safe in the place that you CURRENTLY live?

◯ Not at all
◯ A little bit
◯ Somewhat
◯ Quite a bit
◯ Very much

Does where you currently LIVE make you want to use drugs or drink alcohol?

◯ Not at all
◯ A little bit
◯ Somewhat
◯ Quite a bit
◯ Very much

What is your current or usual work/employment status? (Check all that apply) ([Source: ASI, 5th Ed. 1992])

☐ Unemployed
☐ Employed (full or part-time)
☐ Student
☐ Retired
☐ Homemaker
☐ Disabled
☐ Other

If other employment status, please specify: __________________________________

Closed to Enrollment

How many days in the past month were you paid for working?
(Include "under the table" work, paid sick days and vacation.)

____
([Source: ASI, 5th Ed. 1992])

INJECTION DRUG USE

How many times have you injected drugs in the PAST MONTH?

____

How many days in the last month did you inject opioids?

____
(Enter a numerical value between 0 and 30.)

In the past 3 months, have you had any of the following as a result of your injection drug use?

- ☐ Abscess
- ☐ Bone infection
- ☐ Cellulitis
- ☐ Endocarditis (infection of the heart lining)
- ☐ Other

Specify 'other' health problem from injecting drugs:

____

TOBACCO, ALCOHOL, & DRUG USE

Which of the following statements best reflects your use of (drug used most) at the present time. (Select only one answer)

- ◯ I have stopped using
- ◯ I want to use more
- ◯ I have no desire or intention to stop using
- ◯ I think about not using, but have no specific plan to stop yet
- ◯ I have contacted a treatment agency or taken some other specific step with the intention of stopping my use of

How confident are you in your ability to be completely abstinent (clean) from alcohol and drugs in the next 30 days?
([Source: Brief Addiction Monitor])

- ◯ Not at all
- ◯ Slightly
- ◯ Moderately
- ◯ Considerably
- ◯ Extremely

In the past 30 days, how many days were you in any situation or with any people that might put you at an increased risk for using alcohol or drugs (for example, around risky "people, places or things")
([Source: Brief Addiction Monitor])

- ◯ 0
- ◯ 1-3
- ◯ 4-8
- ◯ 9-15
- ◯ 16-30

Closed to Enrollment

In the past 30 days, how many days did you attend self-help meetings like AA or NA to support your recovery? ([Source: Brief Addiction Monitor])

◯ 0
◯ 1-3 times
◯ 4-8 times
◯ 9-15 times
◯ 16-30 times

How many days in the past month have you used tobacco products or smoked cigarettes? ________________________________ (Days Range 0-30, Enter '0' if you have not used tobacco products in the past month)

How many days in the past month have you drank alcoholic beverages? ________________________________ (Days Range 0-30, Enter '0' if you have not had alcohol in the past month)

How many days in the past month have you used cocaine or crack? ________________________________ (Days Range 0-30, Enter '0' if you have not used cocaine or crack in the past month)

How many days in the past month have you used marijuana or hashish? ________________________________ (Days Range 0-30, Enter '0' if you have not used marijuana in the past month)

How many days in the past month have you used heroin? ________________________________ (Days Range 0-30, Enter '0' if you have not had heroin in the past month)

How many days in the past month did you use prescription medication to get high? ________________________________ (Days Range 0-30, Enter '0' if you have not used prescription pain pills in the past month)

How many days in the past month have you used methamphetamines or amphetamines? ________________________________ (Days Range 0-30, Enter '0' if you have not used meth in the past month)

How many days in the past month have you misused benzodiazepines (anti-anxiety) medications? ________________________________ (Days Range 0-30, Enter '0' if you have not used benzodiazepines or sedatives in the past month)

How many days in the past month have you misused sedative (sleep) medications? ________________________________

In the past month have you misused any other drugs not mentioned? ◯ Yes ◯ No

If yes, please specify the other drug or drugs you've misused. ________________________________

Closed to Enrollment

Physical Health

In general, how would you rate your physical health? ([Source: PROMIS global])

◯ Excellent
◯ Very good
◯ Good
◯ Fair
◯ Poor

In general, would you say your quality of life is:
([Source: PROMIS global])

◯ Excellent
◯ Very good
◯ Good
◯ Fair
◯ Poor

In the past 90 days, has a doctor or health professional told you that you have a problem with your LIVER? ◯ Yes ◯ No

If yes, which of the following liver problems or problem do you have?
(Check ALL that apply.)

☐ Hepatitis A
☐ Hepatitis B
☐ Hepatitis C
☐ Cirrhosis
☐ Other
☐ None of the above

Please specify 'other' liver problem: ______________________________

In the past 90 days, has a doctor or health professional told you that you have a sexually transmitted infection or disease? ◯ Yes ◯ No

If yes, has a doctor or health professional told you that you have any of the following:
(Check ALL that apply.)

☐ Chlamydia
☐ Gonorrhea
☐ Syphilis
☐ Herpes
☐ HPV (genital warts)
☐ HIV/AIDS
☐ Other
☐ None of the above

Specify 'other' STD: ______________________________

In the past 90 days, has a doctor or health professional told you that you have any of these problems?

(Check ALL that apply.)

☐ TBI (Traumatic Brain Injury)
☐ Broken bones
☐ Stroke
☐ Seizures
☐ Other
☐ None of the above

Specify 'other' health problem: ______________________________

Closed to Enrollment

HEALTH SERVICES

Do you currently have health insurance or health care coverage? ◯ Yes ◯ No

In the past 90 days have you been treated in the emergency department (ED) for your ALCOHOL or DRUG use? ◯ Yes ◯ No
(EXCLUDE times that you were in ED for physical health problem(s))

How many times have you been treated in the EMERGENCY DEPARTMENT (ED) in the past 90 days for your ALCOHOL or DRUG use? ________________________________

In the past 90 days, have you been hospitalized (slept overnight) for your ALCOHOL or DRUG use? ◯ Yes ◯ No
(EXCLUDE times that you were in hospital for physical health problem(s))

In the past 90 days, how many nights you been hospitalized for your alcohol or drug use? ________________________________
(NIGHTS IN HOSPITAL |Response must be a number in this range: 0-365)

MENTAL HEALTH

In general, how would you rate your mental health, including your mood and your ability to think? ([Source: PROMIS Global4])

◯ Poor
◯ Fair
◯ Good
◯ Very good
◯ Excellent

In the past 90 days, has a doctor or health professional told you that you have any of the following?

☐ Depression
☐ Bipolar Disorder
☐ Generalized Anxiety Disorder
☐ Panic Disorder
☐ Schizophrenia
☐ Post-traumatic Stress Disorder (PTSD)
☐ Ohter

If other mental health diagnosis, please specify: ________________________________

Over the past 2 weeks, how often have you been bothered by little interest or pleasure in doing things? ([Source: PHQ4])

◯ Not at all
◯ Several days
◯ More than half the days
◯ Nearly every day

Over the past 2 weeks, how often have you been bothered by feeling down, depressed or hopeless? ([Source: PHQ4])

◯ Not at all
◯ Several days
◯ More than half the days
◯ Nearly every day

Closed to Enrollment

In the past 7 days, I felt worthless.
([Source: PROMIS EDDEP04])

◯ Never
◯ Rarely
◯ Sometimes
◯ Usually
◯ Always

In the past 7 days, I felt helpless.
([Source: PROMIS EDDEP06])

◯ Never
◯ Rarely
◯ Sometimes
◯ Usually
◯ Always

In the past 7 days, I felt hopeless.
([Source: PROMIS EDDEP41])

◯ Never
◯ Rarely
◯ Sometimes
◯ Usually
◯ Always

In the past 7 days, I felt sad.
([Source: PROMIS EDDEP17])

◯ Never
◯ Rarely
◯ Sometimes
◯ Usually
◯ Always

Lately I felt cheerful.
([Source: PROMIS NQPPF22])

◯ Never
◯ Rarely
◯ Sometimes
◯ Usually
◯ Always

Mark the statement that best describes how you have been feeling for the past week, including today:
([Source: Beck Scale for Suicidal Ideation])

◯ I have a moderate to strong wish to live
◯ I have a weak wish to live
◯ I have no wish to live

Mark the statement that best describes how you have been feeling for the past week, including today:
([Source: Beck Scale for Suicidal Ideation])

◯ I would try to save my life if I found myself in a life-threatening situation
◯ I would take a chance on life or death if I found myself in a life-threatening situation
◯ I would not take the steps necessary to avoid death if I found myself in a life-threatening situation

In the past 30 days have you had thoughts of killing yourself? ◯ Yes ◯ No
([Source: SITBI-SF])

Closed to Enrollment

In the past 30 days, how many separate times did you have thoughts of killing yourself? (Please give your best estimate.)

____
([Source: SITBI-SF, modified to reflect last 30 days])

When was the LAST time?
([Source: SITBI-SF, modified to reflect time period])

◯ In the past month
◯ In the past week
◯ In the past 24 hours

Have you ever engaged in non-suicidal self-injury? (This is bodily harm that was NOT intended to kill yourself.)

◯ Yes ◯ No
([Source: SITBI-SF])

In the past 30 days, how many times have you engaged in non-suicidal self-harm behavior?

____
([Source: SITBI-SF, modified to reflect time period])

When was the last time you engaged in non-suicidal self-harm behavior?
([Source: SITBI-SF, modified to reflect time period])

◯ In the past month
◯ In the past week
◯ In the past 24 hours

Now I'm going to go through a list of things that people have done to harm themselves. Please let me know which of these you've done, in the past 30 DAYS:
(PLEASE CHECK ALL THAT APPLY)

([Source: SITBI-SF, modified to reflect time period])

☐ cut or carved skin
☐ hit yourself on purpose
☐ pulled your hair out
☐ gave yourself a tattoo
☐ picked at a wound
☐ burned your skin (i.e., with a cigarette, match or other hot object)
☐ inserted objects under your nails or skin
☐ bit yourself (e.g., your mouth or lip)
☐ picked areas of your body to the point of drawing blood
☐ scraped your skin
☐ "erased" your skin to the point of drawing blood
☐ other

On average, for how long have you thought about self-harm before engaging in it?

(DO NOT read responses)
([Source: SITBI-SF])

◯ 0 seconds
◯ 1- 60 seconds
◯ 2-15 minutes
◯ 16-60 minutes
◯ less than one day
◯ 1-2 days
◯ more than two days
◯ wide range (spans > 2 responses)

In the past 30 days, have you received medical treatment for non-suicidal self-harm behavior?

◯ Yes ◯ No
([Source: SITBI-SF, modified to reflect time period])

Closed to Enrollment

In the past 30 days, have you attempted to kill yourself? (check only one response)
([Source: SBQ-R, modified to reflect time period])

◯ Never
◯ It was a brief passing thought
◯ I have had a plan at least once to kill myself but did not try to do it
◯ I have had a plan at least once to kill myself and really wanted to die
◯ I have attempted to kill myself, but did not want to die
◯ I have attempted to kill myself, and really hoped to die

In the past 30 days, how often have you thought about killing yourself?
([Source: SBQ-R, modified to reflect time period])

◯ Never
◯ Rarely (1 time)
◯ Sometimes (2 times)
◯ Often (3-4 times)
◯ Very often (5 or more times)

In the past 30 days, have you ever told someone that you were going to commit suicide or that you might do it?
([Source: SBQ-R, modified to reflect time period])

◯ No
◯ Yes, at one time, but did not really want to die
◯ Yes, at one time, and really wanted to die
◯ Yes more than once, but did not want to do it
◯ Yes, more than once, and really wanted to do it

How likely is it that you will attempt suicide someday?
([Source: SBQ-R])

◯ Never
◯ No chance at all
◯ Rather unlikely
◯ Unlikely
◯ Likely
◯ Rather likely
◯ Very Likely

Closed to Enrollment

SOCIAL RELATIONSHIPS & ACTIVITIES

In general, how would you rate your satisfaction with your social activities and relationships?
([Source: PROMIS Global05])

◯ Poor
◯ Fair
◯ Good
◯ Very good
◯ Excellent

I have someone who will listen to me when I need to talk.
([Source: PROMIS FSE31053x2])

◯ Never
◯ Rarely
◯ Sometimes
◯ Usually
◯ Always

I have someone who understands my problems.
([Source: PROMIS FSE31069x2])

◯ Never
◯ Rarely
◯ Sometimes
◯ Usually
◯ Always

Substance Use Treatment

In the past 90 days, have you ever received treatment or counseling for your use of alcohol or any drug, not counting cigarettes?

◯ No ◯ Yes
(SOURCE: NSDUH 2016)

During the past 90 days, have you received treatment for your use of alcohol or drugs at any of the following?
(source: modified NSDUH, 2016)

☐ A drug or alcohol rehab facility as an outpatient
☐ A drug or alcohol rehab facility as an inpatient
☐ A private doctor's office
☐ A prison or jail
☐ Self-help group (such as Alcoholic/Narcotics Anonymous)
☐ Home, family, friends
☐ School, college
☐ Church/religious/spiritual organization
☐ Mental Health Rehab/intervention as an inpatient
☐ Mental Health Rehab/intervention as an outpatient
☐ Counselor, therapist, psychologist, psychiatrist
☐ Court-mandated/sponsored program
☐ Alcohol/drug class or education
☐ Methadone clinic/program
☐ Buprenorphine clinic/progam
☐ Emergency Department
☐ Other

Other, please specify: ______________________________

Miscellaneous Notes:

Closed to Enrollment

WVU IRB Approval on File
PI: Judith Feinberg
1 Medical Center Drive
Morgantown, WV 26506
Study Coordinator: Amanda Stover
304-293-5861

Referrals to Services (3 Month FU)

You are important. I want to talk to you about some ways that I can help you. I would like to know what I can do to help keep you healthy and safe.

Today's Date: ________________________

Interaction was completed: ◯ By Phone ◯ In Person

ADDICTION SERVICES

Participant was connected with the following services for Addiction: (Please select all that apply).

- ☐ Medication Assisted Treatment (Buprenorphine)
- ☐ Medication Assisted Treatment (Methadone)
- ☐ Medication Assisted Treatment (Naltrexone)
- ☐ Therapy
- ☐ Support Groups
- ☐ Recovery Housing
- ☐ Drop-in Center
- ☐ Other

Where was participant referred for Medication Assisted Treatment? ________________________

Where was participant referred for thearpy? ________________________

Where was participant referred for thearpy? ________________________

What support group/groups was the participant referred to? ________________________

Where was the participant referred for recovery housing? ________________________

What drop in center was the participant referred to? ________________________

What other addiction services was the participant referred to? ________________________

Closed to Enrollment

HARM REDUCTION SERVICES

Participant was connected with the following services for Harm Recuction: (Please select all that apply).

- ☐ Safe Injection Practices/Education
- ☐ Clean Injection Equipment
- ☐ Overdose Prevention Education
- ☐ Naloxone
- ☐ Other

Where was the participant referred for harm reduction services? ________________________

Where was the participant referred for naloxone? ________________________

What other harm reduction services was that participant referred to? __________________________________

INFECTIOUS DISEASE SERVICES

Participant was connected with the following services for Infectious Diseases: (Please select all that apply).

- ☐ Hepatitis C Prevention Education
- ☐ Hepatitis C Testing
- ☐ Hepatitis C Treatment
- ☐ HIV/AIDS Prevention Education
- ☐ HIV/AIDS Testing
- ☐ HIV/AIDS Treatment
- ☐ STI/STD Testing
- ☐ STI/STD Treatment
- ☐ Skin or Soft Tissue Infection Treatment
- ☐ Other

Where was the participant referred for Hepatitis C testing? __________________________________

Where was the participant referred for Hepatitis C treatment? __________________________________

Where was the participant referred for HIV/AIDS testing? __________________________________

Where was the participant referred for HIV/AIDS treatment? __________________________________

Where was the participant referred for STI/STD testing? __________________________________

Where was the participant referred for STI/STD treatment? __________________________________

Where was the participant referred for skin/soft tissue infection treatment? __________________________________

Sepcify other infection disease treatment referal: __________________________________

Where was the participant referred? __________________________________

Closed to Enrollment

MENTAL HEALTH SERVICES

Participant was connected with the following services for Mental Health: (Please select all that apply).

- ☐ Therapy
- ☐ Medication
- ☐ Suicide Prevention/Assessment
- ☐ Evaluation
- ☐ Inpatient Services
- ☐ Crisis Management
- ☐ Other

Where was the participant referred for therapy? __________________________________

Where was the participant referred for medication? __________________________________

Where was the participant referred for suicide prevention services/assessment? __________________________________

Where was the participant referred for mental health evaluation? __________________________________

Where was the participant referred for inpatient mental health services? __________________________________

Where was the participant referred for additional crisis management? __________________________________

Where was the participant referred for other mental health services? __________________________________

PRIMARY CARE SERVICES

Participant was connected with the following services for Primary Care: (Please select all that apply).

- ☐ General Healthcare
- ☐ Chronic Disease Management
- ☐ Vaccinations
- ☐ Acute Illness
- ☐ Women's Health
- ☐ Other

Where was the participant referred for general healthcare services? __________________________________

Where was the participant referred for chronic disease management? __________________________________

Where was the participant referred for vaccinations? __________________________________

What vaccination(s) was the participant interested in receiving? __________________________________

Where was the participant referred for actue illness? __________________________________

Where was the participant referred for women's healthcare? __________________________________

What other primary care services was the participant referred to? __________________________________

SOCIAL SERVICES

Closed to Enrollment

Participant was connected with the following services for Social Services: (Please select all that apply).

☐ Legal Services
☐ Child Care
☐ Insurance
☐ Employment
☐ Transportation
☐ Housing
☐ Clothing
☐ Eye Care
☐ Dental
☐ Food
☐ Emergency Assistance
☐ Abuse Support
☐ Family Services
☐ Schooling/Professional Training
☐ Other

Where was the participant referred for legal services? ________________________

Where was the participant referred for child care/services? ________________________

Where was the participant referred for insurance? ________________________

Where was the participant referred for employment? ________________________

Where was the participant referred for housing? ________________________

Where was the participant referred for clothing? ________________________

Where was the participant referred for eye care? ________________________

Where was the participant referred for dental? ________________________

Where was the participant referred for food assistance? ________________________

Where was the participant referred for emergency assistance? ________________________

Where was the participant referred for abuse suport? ________________________

Where was the participant referred for family services? ________________________

Where was the participant referred for schooling or professional training? ________________________

Where was the participant referred for other social services? ________________________

WVU IRB Approval on File
PI: Judith Feinberg
1 Medical Center Drive
Morgantown, WV 26506
Study Coordinator: Amanda Stover
304-293-5861

Closed to Enrollment
